# Familial Alzheimer’s Disease Neurons Bearing Mutations in *PSEN1* Display Increased Calcium Responses to AMPA as an Early Calcium Dysregulation Phenotype

**DOI:** 10.3390/life14050625

**Published:** 2024-05-12

**Authors:** Helena Targa Dias Anastacio, Natalie Matosin, Lezanne Ooi

**Affiliations:** 1 Molecular Horizons and School of Chemistry and Molecular Bioscience, University of Wollongong, Northfields Avenue, Wollongong, NSW 2522, Australia; htda204@uowmail.edu.au; 2 School of Medical Sciences, Faculty of Medicine and Health, The University of Sydney, Sydney, NSW 2050, Australia; natalie.matosin@sydney.edu.au

**Keywords:** Alzheimer’s disease, induced pluripotent stem cells, neurodegeneration, AMPA, glutamate

## Abstract

Familial Alzheimer’s disease (FAD) can be caused by mutations in *PSEN1* that encode presenilin-1, a component of the gamma-secretase complex that cleaves amyloid precursor protein. Alterations in calcium (Ca^2+^) homeostasis and glutamate signaling are implicated in the pathogenesis of FAD; however, it has been difficult to assess in humans whether or not these phenotypes are the result of amyloid or tau pathology. This study aimed to assess the early calcium and glutamate phenotypes of FAD by measuring the Ca^2+^ response of induced pluripotent stem cell (iPSC)-derived neurons bearing PSEN1 mutations to glutamate and the ionotropic glutamate receptor agonists NMDA, AMPA, and kainate compared to isogenic control and healthy lines. The data show that in early neurons, even in the absence of amyloid and tau phenotypes, FAD neurons exhibit increased Ca^2+^ responses to glutamate and AMPA, but not NMDA or kainate. Together, this suggests that *PSEN1* mutations alter Ca^2+^ and glutamate signaling as an early phenotype of FAD.

## 1. Introduction

Alzheimer’s disease (AD) is the most common neurodegenerative disorder in the world, with memory loss and cognitive dysfunction being common symptoms. The majority of cases are termed sporadic, caused by a combination of genetic and environmental factors, while less than 2% of cases are termed familial Alzheimer’s disease (FAD) [1], caused by mutations in one of three genes: *PSEN1*, *PSEN2,* or *APP*, which each play a role in the generation of amyloid-β peptides [2]. Amyloid-β plaques in the extracellular space [3] and hyperphosphorylated tau in neurons [4] are two of the hallmarks of the disease. Current treatments available alleviate behavioral symptoms but do not stop neuronal degeneration. Hence, there is a great need to identify early pathways altered by the disease.

Calcium (Ca^2+^) dyshomeostasis is implicated in the pathogenesis of AD [5,6]. Several studies have reported mutations in the presenilin genes that alter intracellular Ca^2+^ levels by modifying endoplasmic reticulum (ER) and lysosomal Ca^2+^ release [7,8,9,10,11]. Apart from Ca^2+^ release from intracellular stores, the elevation of cytosolic Ca^2+^ concentration can occur through the influx of extracellular Ca^2+^. The activation of glutamate receptors is a major source of Ca^2+^ influx [5]. Glutamate is the most abundant excitatory neurotransmitter in the mammalian central nervous system, and it mediates excitatory synaptic transmission via activation of ionotropic (iGluRs) and metabotropic (mGluRs) glutamate receptors in the post-synaptic membrane. Ionotropic receptors are ligand-gated ion channels that mediate excitatory synaptic transmission via the influx of Na^+^ and Ca^2+^ into the cytoplasm. They are divided into 3 groups: α-amino-3-hydroxy-5-methyl-4-isoaxazolepropionic acid (AMPA), *N*-methyl-d-aspartate (NMDA), and kainate (KA) receptors [12]. AMPARs are composed of a combination of four subunits (GluA1-GluA4). In the rodent cortex, GluA1 comprises almost 50% of all AMPAR subunits, followed by similar levels of GluA2 and GluA3 [13]. Soluble oligomeric Aβ binds to AMPARs and promotes their internalization, leading to depressed synaptic transmission [14]. However, contrasting results have also found that Aβ increases expression of synaptic AMPAR, increasing synaptic transmission [15]. Hyperphosphorylated tau mislocalised to dendritic spines also disrupts synaptic function by dysregulating AMPAR trafficking [16]. NMDARs have seven different subunits: one GluN1, four GluN2 (GluN2A, GluN2B, GluN2C, and GluN2D), and two GluN3 subunits (GluN3A and GluN3B). Functional NMDARs are heterotetramers composed of two GluN1 and two GluN2 or GluN3 subunits [17]. Activation of extrasynaptic NMDARs increases production of tau [18] and Aβ [19,20]. In turn, amyloid-β increases extrasynaptic NMDAR activation by decreasing neuronal glutamate uptake [21,22,23], but it can also promote NMDAR endocytosis, causing synaptic depression [24]. The NMDAR antagonist memantine is used to treat moderate to severe cases of AD [25], thus targeting NMDA signalling can address some of the symptoms of AD. Kainate receptors (KAR) are composed of a tetrameric assembly of subunits GluK1-5. These receptors are strongly implicated in epilepsy and seizures [26], but little is known about their role in AD. Recent work, however, has found decreased protein expression of the GluK2 subunit and impaired KAR-mediated synaptic transmission in a mouse model of AD [27]. The function and expression of iGluRs play an important role in regulating synaptic plasticity. Through this property, neurons can modulate synaptic strength in response to the intensity and duration of a synaptic input, which is thought to underlie the formation of memory [28]. However, the majority of studies in human *postmortem* brain tissue have shown reduced expression of AMPAR, NMDAR, and KAR at the end stages of the disease, as summarised in [29]. 

Even though glutamatergic neurotransmission is required for synaptic plasticity, excessive activation of excitatory receptors results in excitotoxicity, whereby an elevated influx of Ca^2+^ initiates a signalling cascade that promotes cell death [30]. Hence, glutamatergic stimuli and levels of intracellular Ca^2+^ need to be tightly regulated in order to promote synaptic plasticity and avoid neuronal damage. However, a range of evidence suggests there is significant disruption to Ca^2+^ homeostasis, hyperactive (increased activity of) glutamatergic signalling, and neuronal excitability in AD. (1) Studies using different transgenic animal models expressing mutant *APP*, *PSEN,* and/or *MAPT* genes to model AD pathogenesis have detected elevated basal and stimulus-evoked glutamate release in the entorhinal cortex, dentate gyrus, CA1, and CA3 regions of the hippocampus [31]. (2) Concurrently, various studies have found neuronal hyperactivity in animal and cell models of AD, as well as in patients in the early stages of AD [32]. (3) Clinical studies have also detected a hyperactive phenotype of cortical and hippocampal brain regions in individuals at risk of developing AD, while brain hypoactivity was observed at later stages of the disease [33,34]. (4) Similar abnormalities have been detected in animal and cell models of AD, in which neurons displayed elevated Ca^2+^ signals and increased excitability [35,36,37,38]. Cell studies using induced pluripotent stem cells (iPSCs) from AD patients comprise a smaller fraction of research on neuronal excitability changes in AD but represent a useful tool to assess human cells and the impact of FAD-causing mutations compared to isogenic control lines. 

Altogether, the current literature has identified numerous abnormalities in Ca^2+^ signalling and glutamatergic transmission in AD. Here, we aimed to assess the early calcium phenotypes of FAD neurons to identify whether these may precede AD pathology. In particular, we aimed to quantify differences in the magnitude of Ca^2+^ responses to iGluR agonists in early neurons representing FAD patients. Thus, we treated iPSC-derived excitatory cortical neurons from FAD patients, isogenic controls, and healthy individuals with glutamate, NMDA, AMPA, and kainate and measured the associated Ca^2+^ responses to determine the impact of iGluR agonists on the calcium phenotype in FAD neurons. Our data shows that FAD neurons demonstrate increased calcium responses to AMPA, even in the absence of amyloid and tau phenotypes, highlighting an early phenotype in FAD neurons that may contribute to calcium dysregulation. 

## 2. Materials and Methods

### 2.1. Cell Culture and Neuronal Differentiation: Induced Pluripotent Stem Cell Culture

The induced pluripotent stem cell lines used in this study are described in Table 1. All cell lines were checked for karyotype abnormalities, RNA and protein expression of pluripotency markers, ability to differentiate into 3 germ layers, STR profiling, and genomic DNA sequencing. Experiments using human-derived iPSCs were approved by the University of Wollongong Human Ethics Committee and conducted in accordance with HE13/299 (Wollongong, Australia). To establish iPSCs from frozen stocks, cells were thawed at 37 °C in a water bath and mixed with warm DMEM/F12 medium. The cells were centrifuged for 5 min at 300× *g*, the supernatant was aspirated, and the pellet was resuspended in mTeSR1 (Stem Cell Technologies; Vancouver, BC, Canada) medium. Colonies were then plated into a 60 mm tissue culture dish coated with 0.1 mg/mL Matrigel (Corning; Corning, NY, USA), fed with mTeSR1, and kept in a humidified incubator at 37 °C with 5% CO_2_ and 3% O_2_. The medium was changed every day, and spontaneously differentiated cells were manually removed using a sterile pipette tip. Colonies were passaged every 5–7 days with Mg^2+^/Ca^2+^-free Phosphate Buffered Saline (PBS) (Thermo Fisher Scientific; Waltham, MA, USA) with 0.5 mM Ethylenediaminetetraacetic acid (EDTA; Life Technologies; Carlsbad, CA, USA) (PBS/EDTA). After 3–5 min in PBS-EDTA, colonies were detached by flushing DMEM/F12 into the dish. The medium containing the cells was then transferred into a centrifuge tube and centrifuged for 5 min at 300× *g*. The supernatant was discarded, and colonies were resuspended in 1 mL of mTeSR1 before being plated on a new Matrigel-coated dish.

### 2.2. Neuronal Differentiation

To generate excitatory cortical neurons from induced pluripotent stem cells, a well-characterised protocol combines the use of a lentiviral vector to express the neurogenin-2 (NGN2) transcription factor and drive neuronal differentiation, as well as small molecules inhibiting TGF-β and BMP signalling (SB431542 and LDN193189) to drive neurons towards a forebrain identity [42]. Representative images of the cells and the differentiation timeline are shown in Supplementary Figure S1.

Differentiation of iPSCs to excitatory cortical neurons was performed according to Nehme et al. (2018) [42] with minor modifications. Throughout differentiation, cells were maintained in a humidified incubator at 37 °C with 5% CO_2_ and 3% O_2_. After being thawed from frozen stocks, iPSCs were passaged at least once before being differentiated into neurons. Once iPSCs achieved the desired confluence, they were dissociated to single cells with Accutase (Thermo Fischer Scientific) and counted with a Countess II Automated Cell Counter (Thermo Fischer Scientific) using Trypan Blue (Sigma-Aldrich; Burlington, MA, USA). Single cells were plated at the desired density depending on the assay with mTeSR1 (Stem Cell Technologies) and 10 μM Y-27632 (Focus Bioscience, St Lucia, QLD, Australia) in Matrigel (Corning) and Poly-D-lysine (10–50 μg/mL; Sigma-Aldrich) coated plates. After 24 h, the cells were transduced with lentivirus (1–2 μL virus/1 mL medium) containing rtTA and NGN2-GFP expression vectors. The following day (day 0), the medium was replaced with neuronal differentiation medium containing 1 μg/mL doxycycline (Sigma-Aldrich), 10 μM SB431542 (Focus Bioscience), and 0.1 μM LDN-193189 (Focus Bioscience). On day 1, the culture medium was fully changed to a neuronal differentiation medium containing 1 μg/mL doxycycline, 10 μM SB431542, 0.1 μM LDN-193189, and 0.5 μg/mL puromycin. The same procedure was repeated on days 2 and 3. On day 4, the medium was gradually transitioned to neuronal maturation medium by adding 25% neuronal maturation medium and 75% neuronal differentiation medium containing 10 ng/mL BDNF (Miltenyi Biotec; Gladbach, Germany). The medium was changed every day until neurons were in 100% neuronal maturation medium (day 7). After that, a half-medium change was carried out every other day with neuronal maturation medium containing 1 μg/mL doxycycline and 10 ng/mL BDNF. Doxycycline was maintained until day 10 of differentiation. Neurons were grown until day 35 before experiments were conducted.

### 2.3. Lentivirus Production

Lentiviruses were produced by co-transfecting HEK293T cells (ATCC; Manassas, VA, USA) with two packaging plasmids, one envelope plasmid, and one transfer plasmid (NGN2 or rtTA). Throughout the experiment, the cells were maintained in a humidified incubator at 37 °C with 5% CO_2_ and 3% O_2_. For each lentivirus produced (NGN2 and rtTA), one T-75 flask was plated with 4 × 106 HEK293T cells in DMEM/F12 with 1× Glutamax (Thermo Fischer Scientific) and 5% fetal bovine serum (FBS; Gibco; Grand Island, NY, USA). The following day, 1.5 mL of Opti-MEM was mixed with the plasmid DNAs. On a different falcon tube, 75 μL of polyethylenimine (PEI) transfection reagent (3:1 ratio with DNA) was mixed with 1.5 mL of Opti-MEM (Gibco) and incubated for 5 min. Then, the DNA/Opti-MEM mix was combined with the PEI/Opti-MEM solution and incubated for 20 min to allow the formation of DNA/lipid complexes. This solution was then gently added to the T-75 flasks containing the HEK293T cells. After 5–7 h, the medium was discarded and replaced with fresh DMEM/F12 medium (with 1× Glutamax and 5% FBS). The next day, the medium containing viral particles was collected and stored at 4 °C, and fresh medium was added to the cells. The same procedure was repeated for another two days. After 3 days of viral media collection, the media was combined and centrifuged at 200× *g* for 2 min to pellet and remove HEK293T cells. The viral supernatant was filtered through a 50 mL syringe fitted with a 0.45 μm syringe filter and moved to an ultracentrifuge tube. The tubes were weighted to ensure they were within 0.1 g of each other, and, if necessary, sterile H_2_O was added. The virus was spun at 23,500 rpm for 2 h at 4 °C in an ultracentrifuge. Following centrifugation, the supernatant was discarded and PBS (200× initial volume) was added to the viral pellet, which was gently triturated for approximately 10 min. The viral particles were aliquoted and stored at −80 °C until further use.

### 2.4. Neuronal Characterization by Immunocytochemistry

Cells grown for immunocytochemistry were plated on glass coverslips coated with Matrigel (Corning) or Matrigel and Poly-D-lysine (10–50 μg/mL; Sigma-Aldrich) for iPSCs and neurons, respectively. Cells were washed with TBS and fixed with 4% (*w*/*v*) paraformaldehyde (PFA) in PBS for 20 min, and then washed 3 times with TBS. Cells were permeabilised in 0.3% Triton X-100 in TBS for 10 min at room temperature. Following permeabilisation, cells were blocked in 10% goat serum in TBS for 1 h at room temperature. Then, cells were incubated with primary antibodies (in 10% goat serum in TBS) at specified dilutions overnight at 4 °C. The next day, cells were washed three times in wash buffer (0.1% triton X-100 in TBS) and incubated at room temperature for 1 h with the secondary antibody (in 10% goat serum in TBS) at specified dilutions. After that, cells were washed 3 times in wash buffer and incubated with Hoechst 33342 (1:1000; Thermo Fischer Scientific) in TBS for 15 min at room temperature. Then, cells were washed in TBS, and coverslips were mounted on glass slides using ProLong Gold Antifade Mountant (Thermo Fischer Scientific). Cells were imaged on the Leica TCS SP8 confocal microscope (Leica Microsystems) and analysed with the Leica Application Suite—Advanced Fluorescence (LAS-AF) 2.6.1–7314 software (Leica Microsystems, Wetzlar, Germany). For the no primary antibody control, no primary antibody was added, and both secondary antibodies were added using the same conditions used for MAP2 and GFAP. 

Antibodies: Anti-GFAP Chicken, pAb 1:800 Sigma-Aldrich #ab5541; Anti-MAP2 Mouse, mAb 1:200 Sigma-Aldrich #MAB3418; Anti-Oct4 Mouse, mAb 1:1000 Stem Cell Technologies #01550; Anti-SSEA-4 Mouse, mAb 1:200 Abcam #ab16287; Goat anti-mouse IgG (H + L) Alexa Fluor 488 Goat 1:1000 Thermo Fischer Scientific # A-11001; Goat anti-mouse IgG (H + L) Alexa Fluor 555 Goat 1:1000 Thermo Fischer Scientific # A-21422; Goat anti-chicken IgY (H + L) Alexa Fluor 647 Goat 1:1000 Thermo Fischer Scientific # A-21449.

### 2.5. Amyloid-β Enzyme-Linked Immunosorbent Assay (ELISA)

Aβ40 and Aβ42 levels were measured using an enzyme-linked immunosorbent assay (ELISA) (Thermo Scientific). The conditioned medium from cultured neurons was collected with 1× cOmplete protease inhibitor cocktail (Roche, Switzerland) and 1× PhosphoSTOP phosphatase inhibitor (Roche). Assays were performed following the manufacturer’s instructions. Absorbance at 450 nm was measured on a SpectraMax Plus Microplate Reader (Molecular Devices; San Jose, CA, USA). Each sample was measured in triplicate, and the final protein concentration was determined using a standard curve generated using the Aβ peptide standard provided in the assay kit.

The Aβ40 and Aβ42 enzyme-linked immunosorbent assays (ELISA) (Thermo Fischer Scientific) were performed as per the manufacturer’s instructions.

### 2.6. Western Blotting for Tau: Protein Extraction

Cells were washed twice with 1× TBS and incubated with RIPA buffer (50 mM Tris HCl pH 7.4, 1% Sodium deoxycholate, 150 mM NaCl, 1 mM EDTA, 1% Triton-X and 0.1% SDS in MilliQ) containing 1× cOmplete protease inhibitor cocktail (Roche) and 1× PhosphoSTOP phosphatase inhibitor (Roche) for 20 min on ice. Cell lysate was vortexed for 10 sec and centrifuged at 10,000× *g* for 5 min. The supernatant was transferred into a new microcentrifuge tube and stored at −80 °C until use. 

### 2.7. Detergent Compatible (DC) Protein Assay

The Detergent Compatible Protein Assay (Bio-Rad; Hercules, CA, USA) was used to measure total protein concentration according to the manufacturer’s instructions. A total of 5 μL of sample was mixed with 25 μL of reagent A’ (cell lysate) or A (tissue lysate) and 200 μL of reagent B and incubated for 10 min on an orbital plate rocker (Labnet; Edison, NJ, USA). Each sample was measured in triplicate, and the final protein concentration was determined using a standard curve generated using Bovine Serum Albumin (BSA; Sigma-Aldrich) diluted in either RIPA buffer or lysis buffer. Absorbance was read at 750 nm using a SpectraMax Plus Microplate Reader (Molecular Devices).

### 2.8. Western Blotting

Cell pellets were diluted in RIPA buffer. Samples were mixed with Laemmli buffer (Bio-Rad) containing 5% (*v*/*v*) 2-mercaptoethanol (Sigma-Aldrich). Samples were denatured at 70 °C for 10 min before being loaded into a 4–20% Criterion TGX Stain-Free gel (Bio-Rad). The Precision Plus Protein Dual Colour (Bio-Rad) molecular weight marker was also loaded into the gel. Samples were run in duplicates, and a pooled sample was run on all gels to normalise for variations between gels. Following separation by electrophoresis at 160 V for 50 min in SDS buffer, proteins were transferred to an Immobilon-P PVDF membrane (Merck Millipore; Burlington, MA, USA) at 100 V for 60 min in ice-cold transfer buffer. Total membrane protein was imaged with ChemiDoc MP (Bio-Rad). The membrane was then washed 3× in Tris-Buffered Saline with 0.1% (*v*/*v*) Tween (TBST) and blocked in 5% (*w*/*v*) skim milk or 10% goat serum in TBST for 1 h at room temperature. Incubation with primary antibodies in blocking solution was carried out overnight at 4 °C, and then the membrane was washed 3× for 5 min in TBST before being incubated with secondary antibodies in blocking solution for 1 h at room temperature. Following this step, the membrane was washed 3× for 5 min in TBST and then incubated for 5 min with SuperSignal™ West Pico PLUS Chemiluminescent Substrate (Thermo Scientific). Protein bands were detected by chemiluminescence with Amersham 600 RGB (GE Healthcare; Chicago, IL, USA), and relative densitometry values were quantified using ImageLab (Bio-Rad). Each protein band was normalised to its respective total protein and the mean pool total protein.

Antibodies: Anti-AMPAR1 Rabbit pAb 1:1000 Abcam #ab31232; Anti-AMPAR2 Rabbit mAb 1:2000 Abcam #ab133477; Anti-AMPAR3 Mouse mAb Merck Millipore #MAB5416; Anti-AMPAR4 Rabbit mAb Cell Signalling #8070; Anti-Tau-5 Mouse mAb Abcam #ab80579; Anti-Tau s404 Rabbit mAb 1:2500 Abcam #ab92676; Abbreviations: mAb—monoclonal antibody; pAb—polyclonal antibody; Goat anti-mouse IgG (H + L) HRP Goat 1:5000 Merck Millipore #AP308P; Goat anti-rabbit IgG (H + L) HRP Goat 1:5000 Merck Millipore #AP307P.

### 2.9. Flexstation to Measure Calcium Responses to Glutamate, NMDA, AMPA and Kainate

To study neuronal calcium response, iPSC-derived neurons were grown in a clear bottom 96 Well Black Polystyrene Microplate (Corning) for 35 days. A minimum of 6 wells per cell line were used for each drug treatment (glutamate, NMDA, AMPA, and kainate). A standard bathing solution (SBS; 160 mM NaCl, 2.5 mM KCl, 5 mM CaCl_2_, 1 mM MgCl_2_, 5 mM glucose, and 10 mM HEPES) was used for baseline perfusion of neurons. A 60 mM high potassium (High K^+^, 102.5 mM NaCl, 2.5 mM KCl, 5 mM CaCl_2_, 1 mM MgCl_2_, 5 mM glucose, and 10 mM HEPES) buffer was used as a positive control due to its ability to depolarise neurons and induce a calcium response. To measure neuronal response to iGluR agonists, 100 μM glutamate (Tocris, UK), 100 μM NMDA (Tocris), 100 μM AMPA (Tocris), and 100 μM kainate (Tocris) were prepared in SBS. All buffers and drugs were made on the same day the assay was run and adjusted to have a pH of 7.4 and osmolarity between 310 and 320 mOsm, the same as the conditioned neuronal cultured media.

Neurons were washed with SBS and loaded with 2 µM Fura-2-AM buffer (Thermo Fischer Scientific) and Pluronic F-127 acid (20% *w*/*v* in DMSO; Biotium; Fremont, CA, USA) at 37 °C for 30 min. After incubation, cells were washed again in SBS for 30 min before recording was conducted at 37 °C using a FlexStation 3 Multi-Mode Microplate Reader (Molecular Devices). The fluorescence signal was measured per well, corresponding to the calcium response of the neuronal population grown on each well. Fura-2 fluorescence emission at 510 nm was recorded every four seconds following excitation at 340 and 380 nm. To each well, a total of three solutions (SBS, drug of interest, and High K^+^) were added, and fluorescence was measured. Baseline recordings in SBS were taken for 20 s before the addition of the first compound. Each compound was recorded for 60 s before the addition of the following compound. The drugs glutamate, NMDA, AMPA, or kainate were added after SBS, followed by 60 mM High K^+^. The maximum Ca^2+^ response was calculated from the maximum peak of the Fura-2 AM 340/380 nm excitation ratio normalised to baseline fluorescence (ΔF340/380). Wells that did not respond to high K^+^ were excluded from the analysis. The SoftMax Pro 7 software (Molecular Devices) was used for data acquisition.

### 2.10. RT-qPCR for AMPAR Subunits: RNA Extraction and Purification

After being washed twice in PBS, cells were harvested in TRIsure (Bioline; Cincinnati, OH, USA) and left to incubate for 5 min at room temperature. To each 1 mL of cells in TRIsure, 200 μL of chloroform was added, and the sample was shaken vigorously for 15 s and incubated for 15 min at room temperature. Then, cells were centrifuged at 12,000× *g* for 15 min at 4 °C. The upper aqueous phase (RNA) was transferred to a fresh tube, and 500 μL of isopropanol was added. The samples were incubated for 10 min at room temperature and then centrifuged at 12,000× *g* for 10 min at 4 °C. The supernatant was discarded, and the RNA pellet was washed with 70% ethanol and centrifuged at 7500× *g* for 5 min at 4 °C. The supernatant was removed, and the RNA pellet was air-dried for approximately 30 min. Once dried, the pellet was resuspended in UltraPure DNase/RNase-Free Distilled Water (Thermo Fischer Scientific). The NanoDrop 2000c (Thermo Fischer Scientific) was used to measure nucleic acid concentration and purity.

To remove genomic DNA, the TURBO DNA-free kit (Thermo Fischer Scientific) was used following the manufacturer’s instructions. The RNA was diluted in 50 μL of nuclease-free water, and 1× TURBO DNase buffer and 1 μL TURBO DNase inactivation reagents were added. After the solution was incubated at 37 °C for 30 min, the DNase Inactivation Reagent was added and incubated for 5 min at room temperature. The samples were centrifuged at 10,000× *g* for 90 s, and the supernatant (RNA) was transferred to a fresh tube. RNA concentration was calculated with NanoDrop 2000c. The treated RNA was purified by ethanol precipitation. The RNA was diluted in 200 μL UltraPure DNase/RNase-Free Distilled Water and mixed with 220 μL isopropanol and 20 μL 3 M sodium acetate. After being vortexed, the samples were kept at −80 °C overnight and then centrifuged at 16,000× *g* for 20 min at 4 °C. The supernatant was removed, and the pellets were washed with 70% ethanol before being centrifuged at 16,000× *g* °C for another 2 min. The supernatant was discarded, and the pellet was air dried for approximately 30 min before being resuspended in UltraPure DNase/RNase-Free Distilled Water. The final concentration and purity of mRNA were measured using NanoDrop 2000c.

### 2.11. cDNA Synthesis

Complementary DNA (cDNA) was synthesised using the Tetro cDNA Synthesis Kit (Bioline). The protocol was followed according to the manufacturer’s instructions: 2 μg RNA was mixed with 1 μL oligo (dT)18, 1 μL 10 mM dNTP mix, 1 μL tetro reverse transcriptase enzyme, 4 μL 5× RT buffer, 1 μL RNAse inhibitor, and enough RNAse-free water to complete the 20 μL solution. Samples were incubated for 30 min at 45 °C, followed by 5 min at 85 °C, in the Mastercycler Pro (Eppendorf, Germany). 

### 2.12. qPCR

Primers designed for the coding regions of the genes of interest and primers for housekeeper genes B2M, GAPDH, and HPRT1 were used (Table 2). Each cDNA sample was run in triplicate using 30 ng of cDNA per reaction. No reverse transcriptase RNA control and no template control were run as negative controls. QuantStudio 5 (Thermo Fischer Scientific) was used with the following settings: denaturation at 95 °C for 2 min, followed by 40 cycles of denaturation at 95 °C for 10 s, annealing at a specific primer melting temperature for 10 s, and extension at 72 °C for 10 s. The melt curve protocol started at 95 °C for 5 s, followed by 65 °C for 1 min, ramping up to 97 °C. Primers are listed in Table 2. 

### 2.13. Data Analysis

Data analysis was performed in R v3.3.1 (https://www.r-project.org, accessed on 16 March 2023) using packages ‘library(readxl)’, ‘library(ggplot2)’, ‘library(ggpubr), ‘library(lme4)’, and ‘library(lmerTest)’. The normal distribution of the data was assessed with a Shapiro–Wilk test using the function ‘shapiro.test()’. Data not normally distributed were normalised via logarithmic (log2 or log10), square root (sqrt), or reciprocal (1/x) transformation. For comparison between disease and control groups, the data were subjected to linear regression modelling using the ‘lm()’ function, with independent differentiations included as co-variates.

## 3. Results

### 3.1. Generation of iPSC-Derived Neurons from FAD and Control Lines

Our aim in this study was to specifically assess the early calcium phenotypes of AD, and for this reason, we used day 35 of neuronal maturation, in which cells express neuronal markers and neuronal ion channels but may not yet demonstrate AD pathology. In this well-characterised protocol to generate excitatory cortical neurons, Nehme et al. (2018) [42] demonstrated by RNAseq the expression of AMPAR subunits GluA1-4 over time and found no expression of genes for hindbrain, inhibitory, hypothalamic, diencephalic, hippocampal, dopaminergic, and spinal motor neurons. Single-cell RT-qPCR of day 21 neurons detected mainly markers of cortical upper layer neurons, which was also confirmed by immunostaining and no expression of inhibitory neurons or glia. Furthermore, patch clamping experiments after 28 days of differentiation demonstrated that approximately 75% of the cells recorded were able to fire multiple action potentials [42]. Application of the AMPAR and NMDAR antagonists, NBQX and D-AP5, respectively, showed each compound individually reduced spiking rates and the occurrence of network-wide bursts, confirming the functional expression of AMPAR and NMDARs [42]. On the other hand, the GABA_A_ receptor antagonist, picrotoxin, caused no changes in firing rates or the occurrence of network-wide bursts [42].

To study AD phenotypes in a 2D cell culture model using iPSC-derived neurons, a total of 6 iPSC lines were used, including two familial Alzheimer’s disease cell lines (FAD1 and FAD2) and their respective isogenic controls (IC1 and IC2) and two healthy controls (HC1 and HC2) (Table 1). Each FAD line contains a different mutation in the *PSEN1* gene, which causes early-onset familial Alzheimer’s disease, while the isogenic controls have the same genome as their respective FAD lines, with the exception of the FAD mutation, which has been reverted back to WT. HC1 cells were obtained from a healthy donor with no known family association with FAD1, while the HC2 cell line donor is a healthy family relative of FAD2. Due to their genetic background and family associations, throughout this study, FAD1 neurons were compared against their isogenic control (IC1) and HC1 only, while FAD2 neurons were compared against IC2 and HC2.

To generate iPSC-derived neurons from AD patients, isogenic controls, and healthy individuals, a previously published protocol using small molecules and NGN2 lentivirus was used to generate cortical excitatory neurons [42]. The neurons were differentiated for a total of 35 days prior to analysis.

#### iPSC-Derived Neurons Express Neuronal Marker MAP2

**Figure 1:**
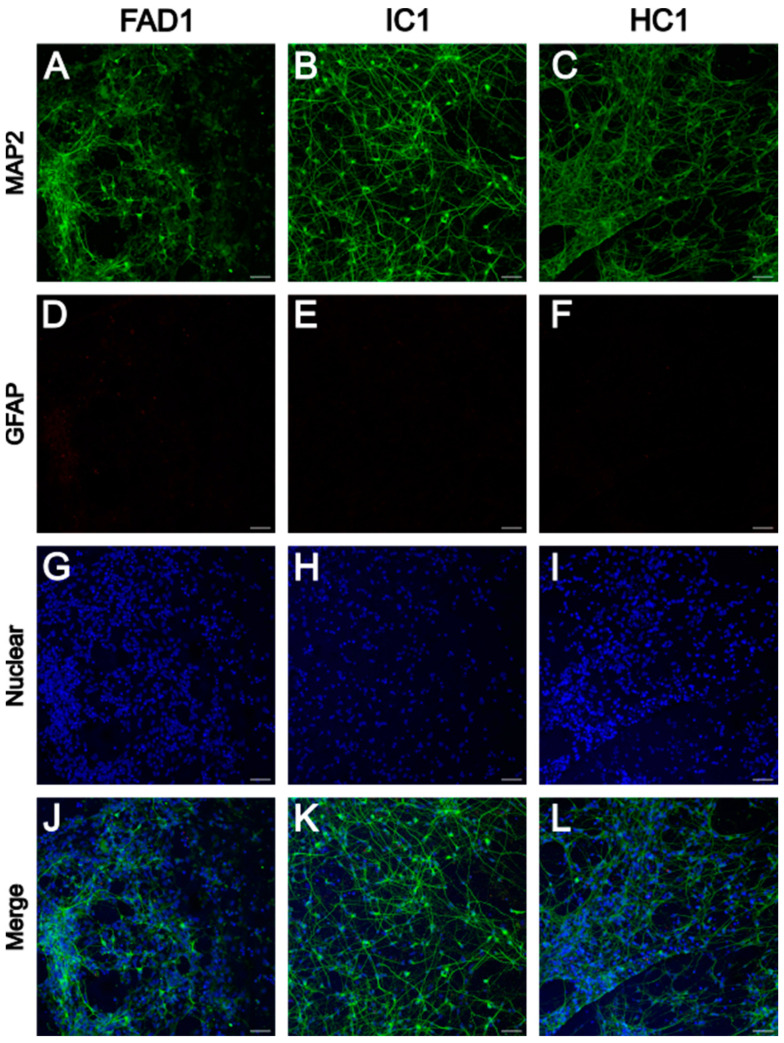
MAP2 and GFAP immunoreactivity in FAD1, IC1, and HC1 iPSC-derived neurons. Representative images showing MAP2 (**A**–**C**), GFAP (**D**–**F**), the nuclear marker Hoechst (**G**–**I**), and the merged image (**J**–**L**) from FAD1, IC1, and HC1. Scale bar = 50 μm. FAD—familial Alzheimer’s disease; HC—healthy control; IC—isogenic control.

**Figure 2:**
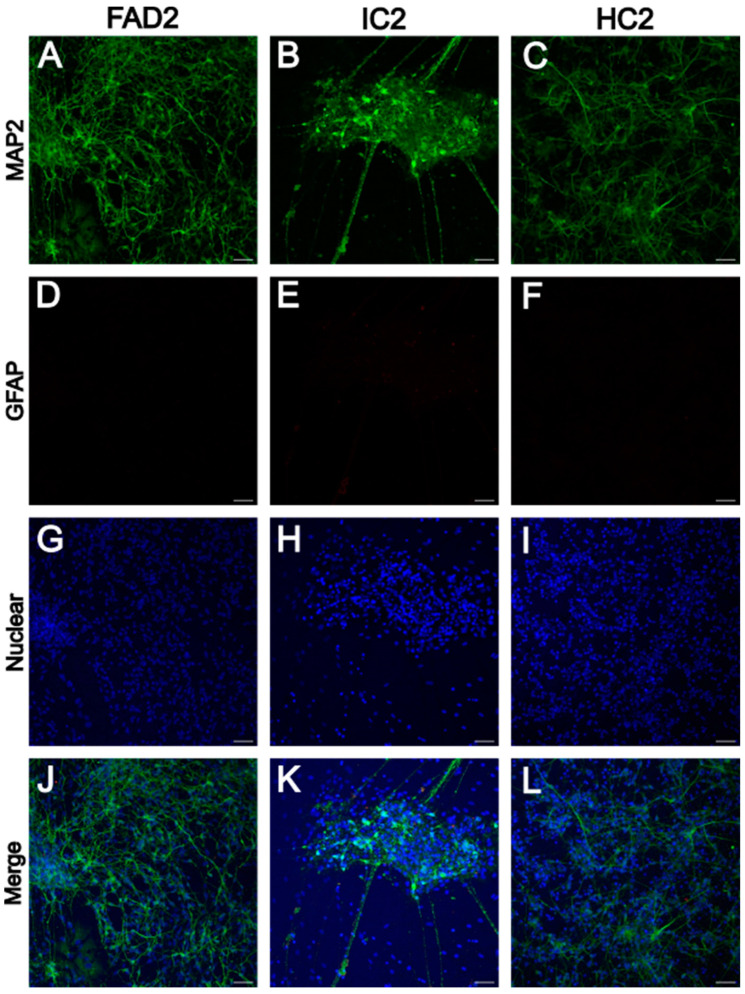
MAP2 and GFAP immunoreactivity in FAD2, IC2, and HC2 iPSC-derived neurons. Representative images showing MAP2 (**A**–**C**), GFAP (**D**–**F**), the nuclear marker Hoechst (**G**–**I**), and the merged image (**J**–**L**) from FAD2, IC2, and HC2. Scale bar = 50 μm. FAD—familial Alzheimer’s disease; HC—healthy control; IC—isogenic control.

To confirm the generation of iPSC-derived neurons after 35 days of differentiation, immunocytochemistry was used. Immunostaining against microtubule-associated protein 2 (MAP2), a neuron-specific cytoskeletal protein, confirmed extensive expression of this marker in all 6 cell lines (FAD1, IC1, HC1, FAD2, IC2, and HC2) (Figure 1 and Figure 2). To check for the presence of glial cells, the cells were also stained against the glial fibrillary acidic protein (GFAP), a type III intermediate filament protein expressed in glial cells. However, no positive staining was observed for GFAP for any of the 6 cell lines (Figure 1 and Figure 2). A non-primary antibody control showed no signal for the MAP2 and GFAP channels (Supplementary Figures S2 and S3). These results confirm the generation of neurons and the absence of GFAP-positive cells after 35 days of differentiation.

### 3.2. FAD NGN2-Derived iPSC Neurons Did Not Show Evidence of Aβ Pathology at Day 35 of Maturation

The decreased Aβ42/Aβ40 ratio in both CSF and plasma is an important biomarker of AD [43,44]. While Aβ40 is the most abundant Aβ peptide, Aβ42 is more prone to aggregation. Due to inter-individual differences in Aβ peptide production, the Aβ42/Aβ40 ratio is considered a more accurate measurement of Aβ pathology than the levels of Aβ42 alone [43,45,46]. 

In 2D in vitro cultures, since Aβ plaques cannot be formed due to the regular medium changes, the elevated production of Aβ42 results in a higher Aβ42/Aβ40 ratio in some long-term cell culture models [36,47,48,49,50]. For example, in day >50–60 neurons, both Aβ and tau pathology can be identified in AD neurons [47,48]. To measure the Aβ42/Aβ40 ratio in disease and control day 35 neurons in this study, an ELISA was performed using medium from neuronal cultures collected at day 35 of differentiation to quantify both Aβ40 and Aβ42 levels separately.

**Figure 3:**
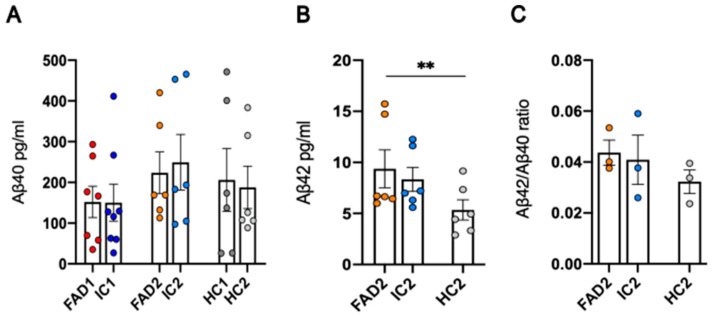
Aβ42/Aβ40 ratio in neuronal cultures. Aβ40 (**A**) and Aβ42 (**B**) levels were measured separately in pg/mL by ELISA and were used for calculation of the Aβ42/Aβ40 ratio (**C**). Data shown are day 35 neurons, n = 2 replicates from 3–4 independent differentiations, and error bars represent ± SEM. For the Aβ42/Aβ40 ratio, data = mean ± SEM, ** *p*< 0.01. Data are normally distributed (FAD2/HC2/IC2 Aβ42/Aβ40 ratio) or normalised to log2 (FAD1/HC1/IC1 Aβ40), sqrt (FAD2/HC2/IC2 Aβ42), or 1/x (FAD2/HC2/IC2 Aβ40) and analysed by linear regression modelling. Independent differentiations were included as co-variates. FAD—familial Alzheimer’s disease; HC—healthy control; IC—isogenic control.

While no significant differences were detected in Aβ40 concentration between disease (FAD1—152.1 ± 38.64, FAD2—223.7 ± 51.23) and isogenic control (IC1—150.1 ± 45.28, *p* = 0.642; IC2—249.6 ± 68.31, *p* = 0.846) or healthy control (HC1—206 ± 77.18, *p* = 0.143; HC2—187.8 ± 52.06, *p* = 0.122) cell lines (Figure 3A), FAD2 (9.37 ± 1.859) neurons showed significantly higher levels of Aβ42 compared to HC2 (5.346 ± 0.994; *p* = 0.001), but no significant differences compared to IC2 (8.333 ± 1.168; *p* = 0.448) (Figure 3B). For the cell lines FAD1, IC1, and HC1, the concentration of Aβ42 in the medium could not be quantified as it was below the detection threshold of the assay, and therefore the Aβ42/Aβ40 ratio could not be calculated. No significant differences were found between the FAD2 (0.044 ± 0.005) and control cell lines (HC2—0.032 ± 0.005, *p* = 0.281; IC2—0.041 ± 0.01, *p* = 0.782) in the Aβ42/Aβ40 ratio (Figure 3C).

These results indicate Aβ40 levels can be detected in the conditioned medium of iPSC-derived neurons, but the levels of Aβ42 may be too low to allow detection by commercial assays. For the group of cell lines in which both Aβ42 and Aβ40 could be measured, FAD1 iPSC-derived day 35 neurons did not show AD-associated changes in Aβ42/Aβ40 ratios.

### 3.3. FAD NGN2 Derived iPSC Neurons Did Not Show Tau Pathology at Day 35 of Maturation

**Figure 4:**
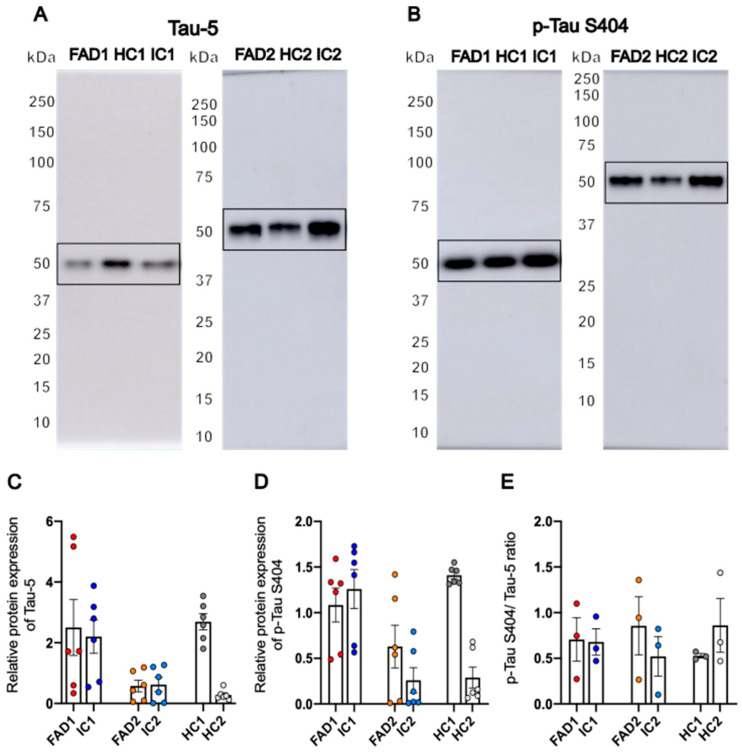
Expression of phosphorylated and total protein tau in neuronal cultures. Representative western blots of Tau-5 (**A**) and p-Tau S404 (**B**) and relative protein expression of Tau-5 (**C**) and p-Tau S404 (**D**) and ratio of p-Tau S404/Tau-5 relative protein expression (**E**). Data shown are day 35 neurons; n = 2 replicates from 3 independent differentiations, normalised to total protein; error bars represent ± SEM. For the p-Tau S404/Tau-5 ratio, data = mean ± SEM. Data are normally distributed (FAD1/HC1/IC1 and FAD2/HC2/IC2 p-Tau S404/Tau-5 ratio and FAD1/HC1/IC1 Tau-5 and p-Tau S404) or normalised to log10 (FAD2/HC2/IC2 p-Tau S404) or sqrt (FAD2/HC2/IC2 Tau-5) and analysed by linear regression modelling. Independent differentiations were included as co-variates; there were no significant differences between groups. FAD—familial Alzheimer’s disease; HC—healthy control; IC—isogenic control; p-Tau—phosphorylated tau.

Intracellular accumulation of misfolded phosphorylated tau (p-Tau) protein is a molecular hallmark of AD that is associated with cognitive decline. Our previous work has shown that day >50–60 AD neurons demonstrate increased phosphorylated tau relative to total tau, consistent with AD-related tau pathology [48]. To assess whether day 35 neurons demonstrate tau phenotypes, we performed western blotting to measure total tau (Tau-5) and phosphorylated tau at serine 404 (p-Tau S404) protein levels between AD and control neurons after 35 days of differentiation (Figure 4A,B). Although tau phosphorylation occurs at different protein epitopes, phosphorylation at serine 404 is one of the earliest events in tau pathology [51], hence why p-Tau S404 was chosen as a marker of AD pathology for this neuronal model of AD. For both Tau-5 and p-Tau S404 western blots, a single band of approximately 50 kDa was observed.

Regarding protein expression of Tau-5, there were no significant differences between FAD (FAD1—2.505 ± 0.923, FAD2—0.575 ± 0.194) and control cell lines (HC1—2.69 ± 0.265, *p* = 0.84; IC1—2.202 ± 0.551, *p* = 0.742; HC2—0.283 ± 0.069, *p* = 0.299; IC2—0.624 ± 0.231, *p* = 0.919) (Figure 4C and Supplementary Figures S4 and S5). Similar results were obtained after analysis of the relative protein amount of p-Tau S404. FAD neurons (FAD1—1.084 ± 0.187, FAD2—0.629 ± 0.234) showed no significant differences compared to their isogenic or healthy controls (HC1—1.41 ± 0.04, *p* = 0.073; IC1—1.259 ± 0.213, *p* = 0.316; HC2—0.29 ± 0.115, *p* = 0.819; IC2—0.259 ± 0.139, *p* = 0.274) (Figure 4D and Supplementary Figures S6 and S7). The ratio of phosphorylated S404 Tau to Tau-5 (total tau) normalised to total protein was calculated to compensate for variations in the production and clearance of tau between cell lines. Data analysis using linear regression modelling showed there were no significant differences between the FAD lines (FAD1—0.706 ± 0.239, FAD2—0.856 ± 0.319) and their respective isogenic (IC1—0.679 ± 0.145, *p* = 0.91; IC2—0.521 ± 0.217, *p* = 0.43) and healthy controls (HC1—0.527 ± 0.024, *p* = 0.465; HC2—0.861 ± 0.294, *p* = 0.99) (Figure 4E).

Overall, both Tau-5 and p-Tau S404 proteins were detected in all cell lines, with no significant differences between FAD and control neurons. These results show that iPSC-derived neurons from FAD donors at day 35 of differentiation did not show the AD-associated phenotype of abnormally elevated S404 tau phosphorylation.

### 3.4. FAD Neurons Demonstrated Increased AMPAR Ca^2+^ Signalling Compared to Isogenic Controls

Dysfunctional calcium signalling and abnormal glutamatergic transmission are common phenotypes of AD. To assess if these phenotypes were recapitulated in iPSC-derived FAD neurons even in the absence of Aβ and tau phenotypes, quantification of calcium responses was conducted in Alzheimer’s disease, isogenic control, and healthy control day 35 neurons. Neurons were treated with 60 mM High K^+^ (to mimic depolarisation), and Ca^2+^ responses were quantified. Ca^2+^ responses were also quantified following the application of 100 µM glutamate, NMDA, AMPA, or kainate in iPSC-derived neurons from FAD patients and controls. Live cell calcium imaging was performed by loading the cells with the ratiometric dye Fura-2 AM prior to treatments. The Flexstation microplate reader was used to measure Ca^2+^ signals from a population of neurons in the wells of a 96 well plate. The maximum Ca^2+^ response from each well was calculated from the maximum peak of the Fura-2 AM 340/380 nm excitation ratio normalised to baseline fluorescence (ΔF_340/380_).

### 3.5. The iPSC-Derived Neurons Displayed Ca^2+^ Responses to High K^+^

**Figure 5:**
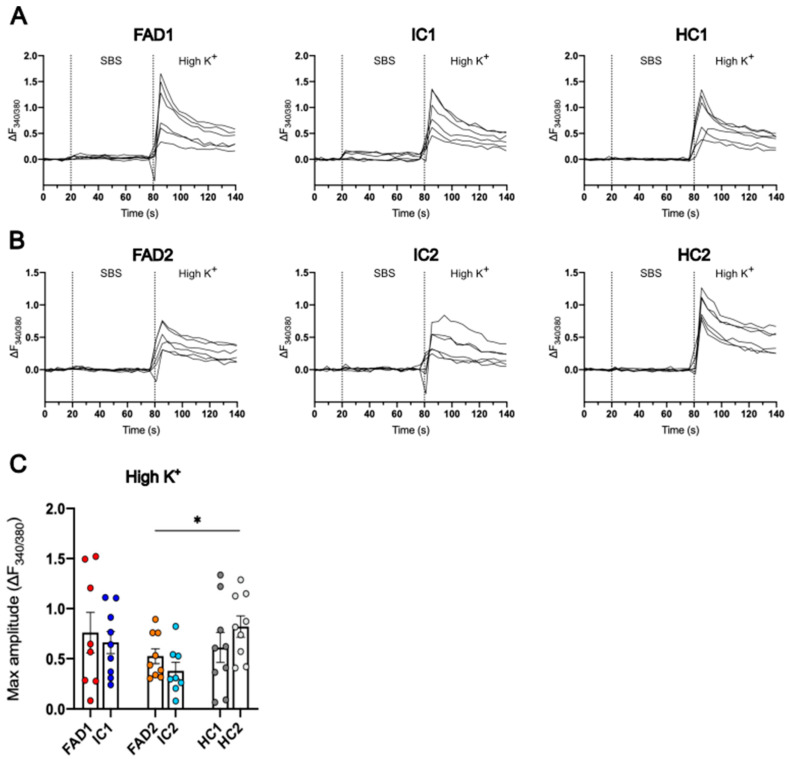
High K^+^ elicits Ca^2+^ responses in iPSC-derived neurons. Representative traces of the change in fluorescence signal ratio over baseline (ΔF_340/380_) in response to 60 mM K^+^ (High K^+^) were recorded from FAD1/IC1/HC1 (**A**) and FAD2/IC2/HC2 neurons (**B**). The maximum Ca^2+^ response to High K^+^ was calculated as the maximum amplitude of the Fura-2 AM fluorescence ratio change over baseline (ΔF_340/380_) (**C**). Day 35 neurons were loaded with the ratiometric dye Fura-2 AM, perfused with SBS, and treated with High K^+^. Data shown are single wells of a 96-well plate containing day 35 neurons, n = 8–15 replicates from three independent differentiations; error bars represent ± SEM; * *p* < 0.05 (FAD2 vs. HC2, but not IC2). Data are normally distributed and analysed using linear regression modelling. Independent differentiations were included as co-variates. FAD—familial Alzheimer’s disease; HC—healthy control; IC—isogenic control.

To verify if day 35 neurons were able to depolarise and produce Ca^2+^ responses, the cells were treated with a high concentration of K^+^ (High K^+^, 60 mM). After loading the neurons with Fura-2 AM, a standard bathing solution (SBS) was added to the cells to allow the measurement of baseline fluorescence, then High K^+^ was applied and Ca^2+^ signals were quantified. Neurons from all cell lines exhibited an increase in the 340/380 fluorescence ratio, indicative of Ca^2+^ responses (Figure 5A,B).

FAD2 neurons (0.525 ± 0.074) had a significantly lower Ca^2+^ response than HC2 cells (0.821 ± 0.107, *p* = 0.026), which displayed the highest maximum amplitude of all cell lines. Although IC2 (0.38 ± 0.083, *p* = 0.272) neurons had the lowest maximum amplitude, this value was not significantly different from the FAD2 line. The peak ΔF_340/380_ of FAD1 (0.760 ± 0.202) cells had no statistically significant difference from IC1 (0.662 ± 0.111, *p* = 0.442) and HC1 (0.612 ± 0.149, *p* = 0.249) cells (Figure 5C).

In sum, iPSC-derived day 35 neurons depolarised and displayed Ca^2+^ signals after treatment with 60 mM K^+^. There were no significant differences in these responses between the FAD and IC neurons. 

### 3.6. FAD Neurons Displayed Increased Ca^2+^ Responses to Glutamate

Glutamate, as the main excitatory neurotransmitter in the CNS, is an important regulator of synaptic plasticity in the brain. However, numerous studies have reported aberrant neuronal excitability in AD patients and lab models of the disease [32]. To investigate how day 35 iPSC-derived neurons respond to glutamatergic stimuli, neurons were treated with 100 µM glutamate, and the Fura-2 AM 340/380 nm excitation ratio normalised to baseline fluorescence (ΔF_340/380_) was recorded over time.

**Figure 6:**
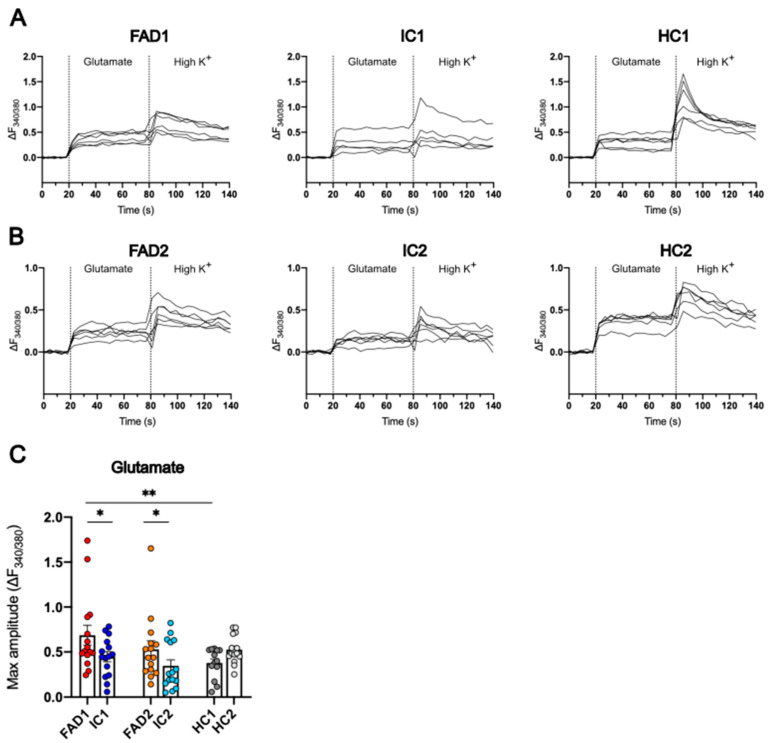
Ca^2+^ responses to glutamate are increased in FAD neurons. Representative traces of the change in fluorescence signal ratio over baseline (ΔF_340/380_) in response to 100 µM glutamate were recorded from FAD1/IC1/HC1 (**A**) and FAD2/IC2/HC2 neurons (**B**). The maximum Ca^2+^ response to 100 µM glutamate was calculated as the maximum amplitude of the Fura-2 AM fluorescence ratio change over baseline (ΔF_340/380_) (**C**). Day 35 neurons were loaded with the ratiometric dye Fura-2 AM, perfused with SBS, and treated with 100 µM glutamate, followed by 60 mM K^+^ (High K^+^). Data shown are single wells of a 96-well plate containing day 35 neurons; n = 8–15 replicates from three independent differentiations; error bars represent ± SEM; * *p* < 0.05, ** *p* < 0.01. Data were normalised to sqrt (FAD1/HC1/IC1) or log2 (FAD2/HC2/IC2) and analysed using linear regression modelling. Independent differentiations were included as co-variates. FAD—familial Alzheimer’s disease; HC—healthy control; IC—isogenic control.

Neurons from all FAD, IC, and HC cell lines displayed Ca^2+^ responses to glutamate (Figure 6A,B). To compare the maximum amplitude of Ca^2+^ responses to glutamate between cell lines, the maximum peak of the Fura-2 AM 340/380 nm fluorescence change (ΔF_340/380_) over baseline was calculated. This analysis confirmed that FAD1 (0.686 ± 0.111) neurons showed significantly higher Ca^2+^ responses compared to IC1 (0.446 ± 0.056, *p* = 0.033) and HC1 (0.377 ± 0.047, *p* = 0.005). Similarly, FAD2 (0.530 ± 0.095) neurons had significantly greater Ca^2+^ responses to 100 µM glutamate than neurons from the relevant isogenic control IC2 (0.345 ± 0.067, *p* = 0.013), but were not significantly different from neurons from the HC2 line (0.526 ± 0.040, *p* = 0.570) (Figure 6C). Thus, FAD iPSC-derived day 35 neurons showed elevated Ca^2+^ signals in response to treatment with 100 µM glutamate compared to their relevant isogenic controls.

### 3.7. FAD Neurons Displayed Increased Ca^2+^ Responses to AMPA but Not NMDA or Kainate Compared to Their Isogenic Control Lines

Glutamate can act on two subgroups of receptors in the post-synapse: mGluRs and iGluRs. The latter are divided into AMPAR, NMDAR, and KAR, and their activation mediates fast excitatory synaptic transmission and modulates synaptic plasticity. To investigate the contribution of specific iGluRs in the elevated Ca^2+^ responses of FAD neurons to glutamate, day 35 iPSC-derived neurons were treated with the AMPAR, NMDAR, and KAR agonists: AMPA, NMDA, and kainate, respectively.

**Figure 7:**
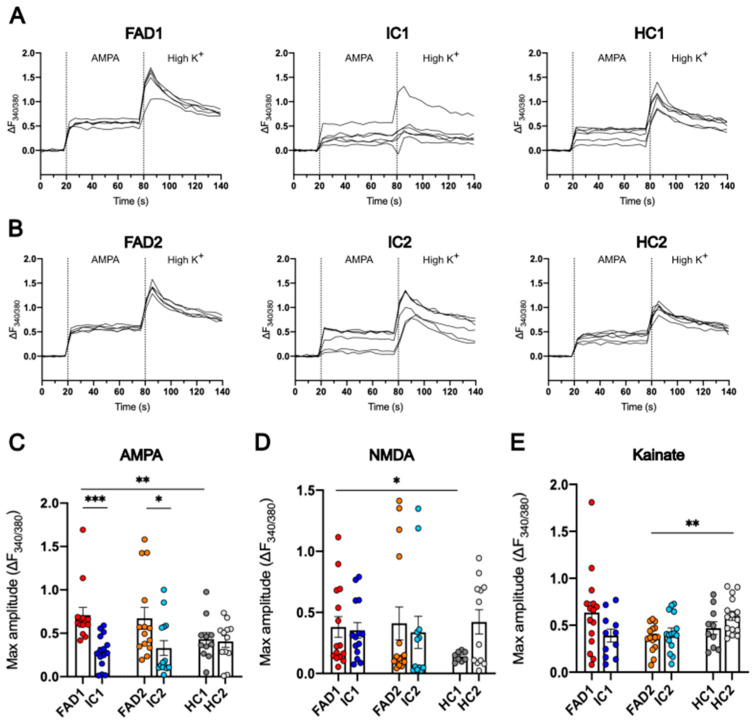
Ca^2+^ responses to AMPA are increased in FAD neurons. Representative traces of the change in fluorescence signal ratio over baseline (ΔF_340/380_) in response to 100 µM AMPA were recorded from FAD1/IC1/HC1 (**A**) and FAD2/IC2/HC2 neurons (**B**). The maximum Ca^2+^ response to 100 µM AMPA (**C**), 100 µM NMDA (**D**), and 100 µM kainate (**E**) was calculated as the maximum amplitude of the Fura-2 AM fluorescence ratio change over baseline (ΔF_340/380_) (**C**). Day 35 neurons were loaded with the ratiometric dye Fura-2 AM, perfused with SBS, and treated with either 100 µM AMPA, 100 µM NMDA, or 100 µM kainate, followed by 60 mM K^+^ (High K^+^). Individual data points are from individual wells of a 96-well plate containing day 35 neurons; n = 8–15 replicates from three independent differentiations; error bars represent ± SEM; *** *p*< 0.001 (AMPA: FAD1 vs. IC1); ** *p*< 0.01 (AMPA: FAD1 vs. HC1; Kainate: FAD2 vs. HC2); * *p*< 0.05 (AMPA: FAD2 vs. IC2; NMDA: FAD1 vs. HC1). Data are normally distributed (FAD2/HC2/IC2 kainate) or normalised to log2 (FAD1/HC1/IC1 kainate and NMDA and FAD2/HC2/IC2 NMDA) or sqrt (FAD1/HC1/IC1 and FAD2/HC2/IC2 AMPA) and analysed using linear regression modelling. Independent differentiations were included as co-variates. FAD—familial Alzheimer’s disease; HC—healthy control; IC—isogenic control.

Tracking of the 340/380 fluorescence change (ΔF_340/380_) over time showed all 6 cell lines responded to 100 µM AMPA (Figure 7A,B). Statistical analysis of the maximum amplitude of ΔF_340/380_ showed both FAD1 (0.709 ± 0.088) and FAD2 (0.673 ± 0.125) exhibited higher Ca^2+^ responses to 100 µM AMPA than their respective isogenic controls, IC1 (0.289 ± 0.048, *p* = 1.53 × 10^−5^) and IC2 (0.330 ± 0.086, *p* = 0.010). FAD1 also had increased Ca^2+^ responses to 100 µM AMPA compared to HC1 (0.432 ± 0.075, *p* = 0.017); however, FAD2 and HC2 (0.406 ± 0.067, *p* = 0.077) were not significantly different (Figure 7C). 

Treatment with 100 µM NMDA showed no significant differences in Ca^2+^ responses from FAD1 (0.380 ± 0.085) and FAD2 (0.41 ± 0.134) relative to their isogenic controls, IC1 (0.353 ± 0.064, *p* = 0.874) and IC2 (0.337 ± 0.132, *p* = 0.692), respectively. However, FAD1 (0.38 ± 0.085) Ca^2+^ signals were significantly elevated compared to HC1 (0.139 ± 0.014, *p* = 0.046), while no significant differences were observed between FAD2 and HC2 (0.422 ± 0.1, *p* = 0.307) (Figure 7D). 

Analysis of Ca^2+^ signals following 100 µM kainate treatment revealed no significant differences between the FAD (FAD1 = 0.636 ± 0.111; FAD2 = 0.37 ± 0.037) lines and their isogenic controls (IC1 = 0.39 ± 0.069, *p* = 0.128; IC2 = 0.42 ± 0.051, *p* = 0.446). Additionally, FAD1 and HC1 (0.47 ± 0.067, *p* = 0.604) had no significant differences. Nonetheless, HC2 (0.601 ± 0.049, *p* = 0.001) neurons had greater Ca^2+^ responses to 100 µM kainate compared to FAD2 (0.37 ± 0.037) (Figure 7E).

Taken together, these data confirm that day 35 iPSC-derived neurons respond to excitatory stimulus and that FAD neurons display abnormally elevated Ca^2+^ signals when treated with glutamate and, more specifically, the AMPAR agonist AMPA, but not with NMDA or kainate. This suggests FAD neurons demonstrate a glutamatergic Ca^2+^ signalling phenotype, contributed (at least in part) by increased AMPAR signalling.

### 3.8. Regulation at the Level of mRNA or Protein of the AMPA Receptor Subunits Does Not Explain the Increased Calcium Responses to AMPA in FAD Neurons Compared to Isogenic Control Neurons

**Figure 8:**
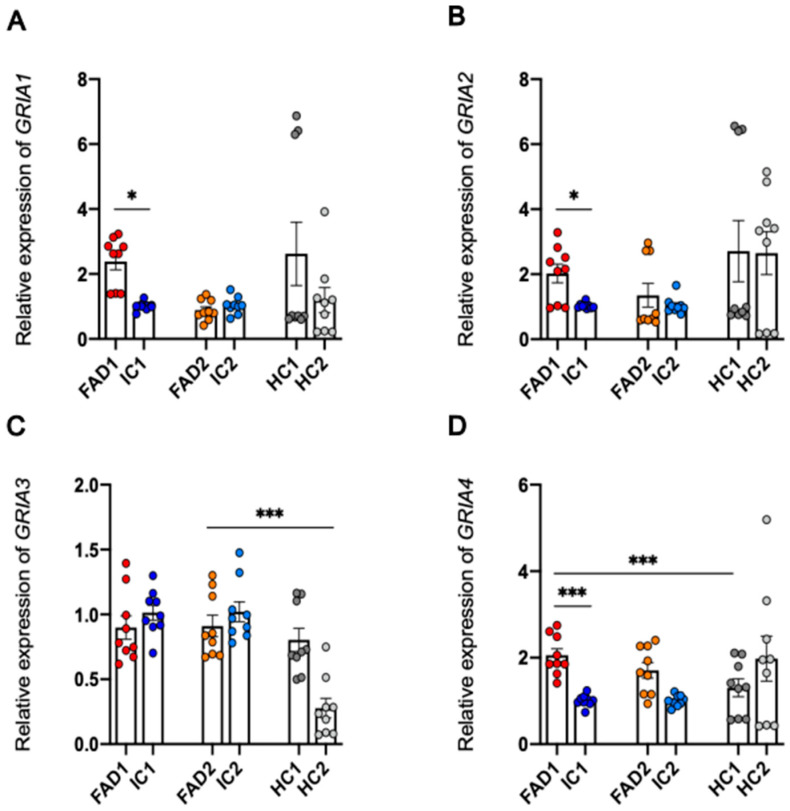
AMPAR subunits mRNA expression of FAD and control neurons. RT-qPCR analysis of GRIA1 (**A**), GRIA2 (**B**), GRIA3 (**C**), and GRIA4 (**D**) mRNA expression of day 35 neurons normalised to housekeeper genes GAPDH, HPRT1, and B2M. Data shown are replicates from one experiment using cDNA collected from three independent differentiations run in triplicate; error bars represent ± SEM; * *p* < 0.05, *** *p*< 0.001. Data are normally distributed (FAD1/HC1/IC1 GRIA3 and GRIA4 and FAD2/HC2/IC2 GRIA3) or normalised to log2 (FAD1/HC1/IC1 GRIA1 and FAD2/HC2/IC2 GRIA1, GRIA2 and GRIA4) or 1/x (FAD1/HC1/IC1 GRIA2) and analysed using linear regression modelling. Independent differentiations were included as co-variates. FAD—familial Alzheimer’s disease; HC—healthy control; IC—isogenic control.

Since the FAD neurons exhibited significantly increased maximum Ca^2+^ responses to AMPA, we next investigated whether changes in mRNA or protein levels of the AMPAR subunits may be responsible for the increased signalling. To test this hypothesis, RT-qPCR was performed to investigate if differences observed in neuronal Ca^2+^ responses to AMPA between FAD and isogenic control neurons were due to differences in mRNA expression of AMPAR subunits 1–4 encoded by the genes *GRIA1*, *GRIA2*, *GRIA3,* and *GRIA4* (Figure 8A–D). 

Expression of *GRIA1* mRNA was significantly higher in FAD1 (2.386 ± 0.257) than IC1 (1.007 ± 0.43, *p* = 0.011) in day 35 neurons, while there was no significant difference between FAD2 (0.888 ± 0.105) and IC2 (1.03 ± 0.087; *p* = 0.527). FAD1 and FAD2 *GRIA1* mRNA expression showed no significant differences from HC1 (2.622 ± 0.977; *p* = 0.132) and HC2 (1.197 ± 0.389, *p* = 0.795), respectively.

Similar results were obtained from the analysis of *GRIA2* gene expression. FAD1 (2.024 ± 0.284) had significantly greater mRNA levels of *GRIA2,* compared to IC1 (1.028 ± 0.028; *p* = 0.027), but was not significantly different from HC1 (2.712 ± 0.941; *p* = 0.116). Although FAD2 (1.353 ± 0.364) had a higher mean mRNA expression of *GRIA2* compared to IC2 (1.039 ± 0.084; *p* = 0.975), this difference did not reach statistical significance. FAD2 was also not significantly different from HC2 (2.649 ± 0.66, *p* = 0.437).

Regarding the relative expression of *GRIA3* mRNA, the only significant difference observed was between FAD2 (0.911 ± 0.082) and HC2 (0.277 ± 0.075, *p* = 2.34 × 10^−6^), with FAD2 neurons displaying higher expression of *GRIA3*. No significant differences were detected between FAD1 (0.899 ± 0.09) and IC1 (1.014 ± 0.058; *p* = 0.24) or HC1 (0.804 ± 0.089, *p* = 0.325) or between FAD2 and IC2 (1.021 ± 0.077, *p* = 0.293).

Lastly, RT-qPCR analysis of *GRIA4* showed FAD1 (2.056 ± 0.153) mRNA levels were significantly higher than both IC1 (1.009 ± 0.045; *p* = 3.38 × 10^−7^) and HC1 (1.303 ± 0.207, *p* = 3.85 × 10^−5^). For FAD2 (1.708 ± 0.182), even though there was a trend towards higher mRNA than IC2 (1.007 ± 0.043, *p* = 0.065), this difference was not statistically significant. There was no significant difference between FAD2 and HC2 (1.977 ± 0.522; *p* = 0.563).

Altogether, these results showed FAD1 iPSC-derived day 35 neurons had increased *GRIA1*, *GRIA2,* and *GRIA4* mRNA expression compared to IC1, but there was no difference in *GRIA3* expression. Furthermore, no significant differences between FAD2 and IC2 were observed in *GRIA1*, *GRIA2*, *GRIA3,* or *GRIA4 mRNA* expression, suggesting that transcriptional regulation of the GRIA genes cannot explain the increased calcium responses to AMPA.

#### Protein Expression of GluA1 and GluA2 Is Not Significantly Different between FAD and Control Neurons

Following the observed elevation in Ca^2+^ responses of FAD neurons compared to their IC neurons when treated with both glutamate and AMPA, it was hypothesised that these differences could be due to a difference in AMPAR subunit protein expression. We focused on GluA1 and GluA2 as the major AMPAR subunits in excitatory neurons. To investigate this hypothesis, total protein extraction from whole cell lysates of day 35 neurons was prepared for western blotting assays. 

**Figure 9:**
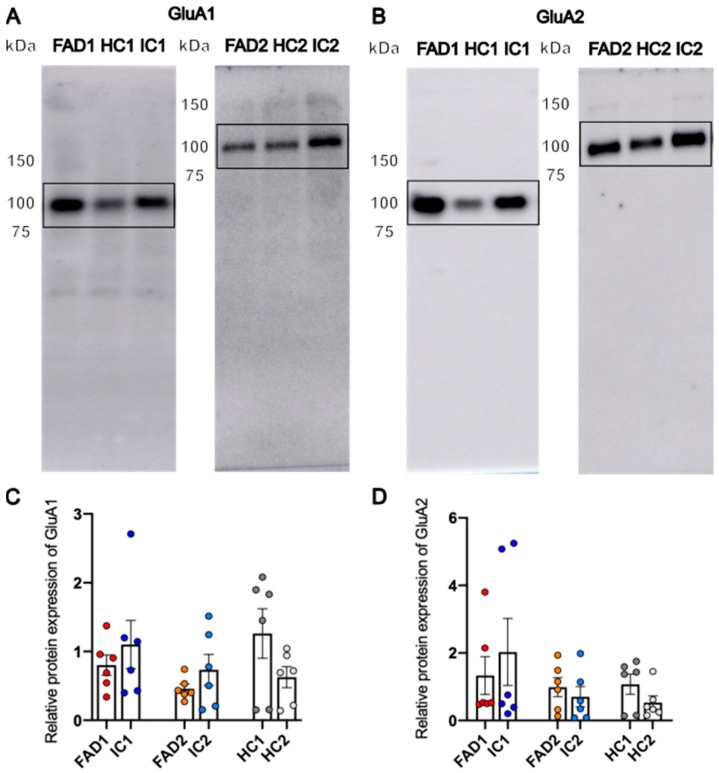
GluA1 and GluA2 protein expression of FAD and control neurons. Representative western blots (**A**,**B**) and relative protein expression of GluA1 (**C**) and GluA2 (**D**). Data shown are day 35 neurons; n = 2 replicates from 3 independent differentiations, normalised to total protein; error bars represent ± SEM. Data are normally distributed (FAD2/HC2/IC2 GluA1) or normalised to log2 (FAD1/HC1/IC1 and FAD2/HC2/IC2 GluA2) or sqrt (FAD1/HC1/IC1 GluA1) and analysed using linear regression modelling. Independent differentiations were included as co-variates; there were no significant differences between groups. FAD—familial Alzheimer’s disease; HC—healthy control; IC—isogenic control.

Western blots for all cell lines detected a strong band of approximately 100 kDa for both GluA1 and GluA2 (Figure 9A,B and Supplementary Figures S8–S11), present at their expected molecular weight. For some cell lines, a band of higher molecular weight, approximately 250 kDa, was also observed, which was not included in the quantification. Densitometry values of the bands at 100 kDa were normalised to respective total protein signals and a mean pooled sample, which were used to account for variability in protein loading and to normalise between separate blots, respectively. Linear regression analysis revealed no significant differences in GluA1 or GluA2 protein expression between FAD (FAD1—0.802 ± 0.148; FAD2—0.46 ± 0.066) and control cell lines IC (IC1—1.1 ± 0.35, *p* = 0.515; IC2—0.733 ± 0.226, *p* = 0.25) and HC (HC1—1.261 ± 0.36; *p* = 0.4; HC2—0.626 ± 0.152, *p* = 0.479) (Figure 9C,D).

These results indicate that the alterations in AMPAR Ca^2+^ signalling in FAD and control iPSC-derived neurons differentiated for 35 days were not due to differences in protein expression of GluA1 and GluA2 AMPAR subunits.

## 4. Discussion

Glutamate and calcium dyshomeostasis have been documented in Alzheimer’s disease; however, it has been difficult to determine whether or not calcium and glutamate phenotypes precede amyloid and tau phenotypes in humans. To address this question in a 2D human cell model of the early stages of the disease, this study aimed to generate iPSC-derived neurons from FAD patients, isogenic controls, and healthy controls and measure Ca^2+^ responses following the application of glutamate, NMDA, AMPA, and kainate. The results obtained in this study showed that day 35 FAD iPSC-derived neurons did not demonstrate evidence of AD-associated Aβ and tau pathology and yet displayed altered Ca^2+^ responses. Neurons from FAD patients had elevated Ca^2+^ responses to both glutamate and AMPA but not to kainate and NMDA when compared to their isogenic controls. Together, the data suggest that mutations in *PSEN1* cause increased Ca^2+^ responses to AMPA as an early phenotype of AD.

### 4.1. FAD Neurons Lacking Aβ and Tau Pathology Show Elevated Ca^2+^ Responses to Glutamate and AMPA Compared to Isogenic Controls

In AD, cortical brain regions are severely affected by both Aβ and tau pathology and, eventually, neuronal loss. Hence, cortical glutamatergic neurons represent an appropriate model to study FAD neuronal calcium responses to excitatory stimuli.

The impact of FAD-causing mutations in the PSEN1 gene, PSEN1^S290C^ and PSEN1^A246E^, was investigated using iPSC-derived neurons from FAD patients as well as isogenic and healthy controls. Although the PSEN1^S290C^ mutation results in the deletion of exon 9, preventing the endoproteolysis of PSEN1, PSEN1^S290C^ is still generally considered to be functionally active, leading to an increased Aβ_42_/Aβ_40_ peptide ratio [52,53], similar to PSEN1^A246E^ [52,54]. This has been suggested to occur due to the ability of PSEN1^S290C^ to adopt a conformation similar to the heterodimeric active PSEN1 as opposed to the inactive PSEN1 holoprotein [55]. Furthermore, the pathologic function of PSEN1^S290C^ in generating elevated Aβ_42_ levels is believed to be independent of its inability to undergo endoproteolysis but rather due to the introduction of the point mutation (S290C), which occurs as a result of the exon 9 deletion [53]. Hence, although PSEN1^S290C^ is structurally different from PSEN1^A246E^, it is not clear whether this results in different AD-associated phenotypes. 

In this study, iPSC-derived neurons were differentiated for a total of 35 days. Changes in cell morphology from iPSCs into neurons and expression of the fluorescent reporter GFP (co-expressed with NGN2) could be observed from day 1 of neuronal differentiation. Immunocytochemistry results from this study confirmed the cell cultures generated expressed the neuronal marker MAP2, while no cells positive for the astrocytic marker GFAP were observed.

The presence of amyloid-β plaques and neurofibrillary tangles in human *postmortem* brain tissue provides the criteria for the neuropathologic diagnosis of AD [56]. Both plasma and cerebral spinal fluid levels of Aβ are decreased in AD and are inversely associated with plaque burden [43,44]. Since Aβ plaques cannot be formed in 2D neuronal cultures due to the frequent media changes, the levels of Aβ peptides released into the cell medium are commonly used as a measurement of Aβ pathology. Several long-term 2D cell culture models of AD have detected elevated Aβ42/Aβ40 ratios [36,47,48,49,50] or increased secreted Aβ42 in the media [47], while others have found no differences [36]. This discrepancy may be explained by the use of different protocols to generate neuronal subtypes or by the length of time that neurons are cultured/matured for. Another important contributing factor is the use of cell lines harbouring different FAD mutations or sporadic AD lines bearing different genetic risk factors and their comparison with either healthy or isogenic controls. Statistical analysis of Aβ42 levels for day 35 iPSC-derived neurons generated for this work showed FAD2 neurons had higher levels of Aβ42 than a healthy control line HC2 but, critically, was not statistically different from its isogenic control IC2. Since healthy controls have an entirely different genome from a familial disease cell line, differences found between these cells may be biased by factors other than the disease-associated mutation and, therefore, complicate the interpretation of the results. Isogenic controls, on the other hand, should have the same genome as their parental cell line, except for the disease-causing mutation that is reverted to WT, making it a more useful comparison to understand the contribution of specific mutations to disease phenotypes. Thus, we expect to observe differences in responses between unrelated lines. Nevertheless, the inclusion of additional healthy controls is helpful to assess variations in responses. Apart from the different types of controls used, the presence of glial cells in the culture can also affect the Aβ phenotype, as they are involved in the clearance of this peptide [57]. Although our iPSC-derived neuronal model did not show any GFAP+ cells after immunostaining analysis, it cannot be ruled out that GFAP-negative astrocytic or other glial populations may be present in the culture and impact Aβ processing. 

The microtubule-associated protein tau is enriched in the axons of mature neurons; however, in pathological conditions such as AD, it is hyperphosphorylated and accumulates in the cell soma and dendrites, where it forms insoluble aggregates and neurofibrillary tangles. A total of 59 tau phosphorylation sites have been detected in human *postmortem* brain tissue of AD patients [58,59], and serine 404 (p-Tau S404) is considered one of the first epitopes to be phosphorylated in the disease [51]. Tau phosphorylation at different epitopes has been previously replicated in cell models of AD, including p-Tau S404 [48], hence its inclusion as a measurement for early stages of tau pathology. However, statistical analysis revealed no significant differences in the levels of p-Tau S404 between FAD and control cell lines in day 35 neurons. Although a combination of factors, such as lipid metabolism, endocytosis, and the immune response, contribute to pathologic tau accumulation [60], elevated Aβ levels are also believed to accelerate tau pathology [61]. Given that an Aβ pathology phenotype at this stage of neuronal maturation was not observed in the FAD neurons, this may explain the absence of a tau phenotype as well. Therefore, neither Aβ accumulation nor increased tau phosphorylation were detected in day 35 FAD iPSC-derived neurons in this study. Thus, this model permits the study of early FAD phenotypes associated with *PSEN1* mutations in human cells. 

Another phenotype of AD is neuronal hyperexcitability (increased excitability), which is thought to precede neuronal hypoexcitability (reduced excitability) and cell death [32]. This elevated neuronal activity is thought to promote neurodegeneration in specific neurons [30] and correlate with cognitive decline. Numerous abnormalities have been suggested to contribute to the change in neuronal excitability, including calcium and glutamate dyshomeostasis [32]. To understand whether there is a calcium phenotype in early FAD neurons, it was first confirmed that all cell lines responded to high K^+^. HC2 neurons demonstrated greater responses to high K^+^ than FAD2 neurons, suggesting HC2 may have a greater density of K^+^ channels compared to FAD2 cells. This could impact neuronal differentiation and excitability, key functions of K^+^ channels [62,63]. Nevertheless, no significant differences were found between the isogenic control IC2 and FAD2 neurons, suggesting the differences observed between HC2 and FAD2 lines originate from genetic differences between the cell lines unrelated to the FAD mutation. Then, to investigate how neurons respond to excitatory stimuli, the experiments aimed to measure neuronal calcium signals after treatment with glutamate and the iGluR agonists, NMDA, AMPA, and kainate. Analysis of the maximum amplitude of the calcium response to each drug revealed FAD neurons had significantly higher calcium responses to both glutamate and AMPA compared to their isogenic controls. No significant differences were observed between the disease and isogenic controls following treatment with NMDA or kainate. Although this work focused on iGluR agonists, the differences in Ca^2+^ responses to glutamate could also stem from changes in mGluR, previously shown to have altered expression and/or activity in AD [64]. Hence, future work is crucial to elucidate the contribution of mGluR to Ca^2+^ dyshomeostasis in the model studied here.

Both Aβ and tau contribute to calcium dyshomeostasis and AMPAR trafficking dysfunction, resulting in a reduction in AMPAR expression and function and defective synaptic plasticity, as reviewed in [65,66]. Nevertheless, the data identified an aberrant calcium signalling phenotype that appears to occur independently of Aβ or tau phenotypes. Consequently, in the absence of evidence for Aβ and early phosphorylated tau S404 changes, alternative mechanisms need to be considered. Notch-1 expression is altered in neurons bearing these same *PSEN1* mutations [67]. Notch signalling is implicated in stem cell renewal, proliferation, and differentiation, as well as neuronal development [68]. Thus, reduced Notch-1 expression in FAD neurons could lead to differential regulation of AMPAR expression in FAD, control iPSC-derived neurons, and explain the phenotypes observed here.

### 4.2. Aberrant Ca^2+^ Signalling of FAD Neurons Occurs Independently of Changes in GluA1 and GluA2 Protein Expression

While the data failed to demonstrate alterations in GluA1 and GluA2 protein levels in FAD compared to isogenic control neurons, there were some increases in *GRIA1*, *GRIA2,* and *GRIA4* at the mRNA level. The transcriptional regulatory mechanisms were not assessed since the transcriptional up-regulation did not lead to concomitant increases in protein levels. AMPAR are tetrameric assemblies of GluA1-GluA4 subunits that permit Na^+^ and Ca^2+^ influx into the cells. Ca^2+^-permeable AMPAR is important for long-term potentiation (LTP) induction and long-term memory formation [69]. The permeability of AMPARs to Ca^2+^ depends on subunit composition, and GluA2 subunit permeability to Ca^2+^ is regulated by RNA editing. The post-transcriptional modification of *GRIA2* RNA from a codon encoding glutamine (Q) to a codon encoding arginine (R) renders this subunit impermeable to Ca^2+^. Thus, AMPARs lacking GluA2 or containing the unedited version of GluA2 are Ca^2+^ permeable [70]. The unedited *GRIA2* mRNA has very low expression in the adult human brain, comprising less than 10% of *GRIA2* in the white matter and less than 1% in the grey matter [71]. In spite of its low expression, unedited GluA2 can contribute to synaptic plasticity and excitotoxic neuronal cell death, as reviewed in [72] Interestingly, lower RNA editing of GluA2 has been reported in AD brains [73,74,75]. Although RT-qPCR of *GRIA2* performed in this study is insufficient to discern edited from unedited *GRIA2*, previous studies have employed different strategies, such as restriction enzyme digestion and Sanger sequencing of PCR products [76,77], to quantify unedited *GRIA2* mRNA. Future work utilising these assays could inform whether changes in the amount of edited or unedited *GRIA2* occur in FAD neurons. 

The expression of AMPAR subunits varies across regions of the mammalian brain. In murine hippocampal synapses, the majority of AMPARs are comprised of GluA1/2 subunits, followed by a smaller fraction of GluA2/3 heterodimers [78,79]. In the adult rat cortex, GluA1 is the predominant subunit (~45% of total AMPAR), followed by GluA2 (21%), GluA3 (27%), and very low levels of GluA4 (less than 6%) [13]. Several studies have shown GluA1 and GluA2 protein expression is decreased in AD *postmortem* brain tissue, as summarised in [29]. Lower expression of GluA1 has been reported in the frontal cortex [80,81,82] and cerebellum [83] of AD brains, while no differences were observed in the temporal cortex compared to healthy individuals [69], suggesting there is a brain region-specific phenotype. Regarding GluA2, this AMPAR subunit has been the most extensively studied in AD. A recent compilation of 38 proteomic studies of AD reported a total of 12 studies where GluA2 protein expression was reduced in the frontal, entorhinal, and parahippocampal cortex, as well as the hippocampus and precuneus brain regions of AD brains [29]. Most of the studies (7 out of 12) found these changes in the frontal cortex. Nonetheless, contrasting results have been reported by a couple of studies that detected an upregulation of GluA2 protein expression in the temporal cortex and hippocampus of AD patients [69,84]. The work conducted by Barbour and colleagues [69] is the only study to examine GluA2 expression in the temporal cortex, indicating GluA2 could be differentially regulated in specific brain regions; however, more studies analysing this area of the brain are required to confirm these results. While Stepler et al. [85] found decreased levels of GluA2 in tissue homogenates of the whole hippocampus, Yeung et al. [84] found GluA2 expression was elevated in a subregion of the hippocampus, the stratum moleculare layer of the dentate gyrus, but no significant changes were detected in the stratum granulosum and hilus areas of the dentate gyrus, or in any of the CA1, CA2, or CA3 regions of the hippocampus. Also, no changes were observed in the superior temporal gyrus, subiculum, and entorhinal cortex of AD versus control donors. Thus, a potential explanation for these discrepancies is that GluA2 expression is differentially altered in specific subregions of the hippocampus. Furthermore, although the *postmortem* brain tissue used by Yeung et al. [84] was obtained from a brain bank located in New Zealand, the ethnicity of their cohort was not specified. In contrast, Stepler et al. [85] analysed brains from African American and non-Hispanic White individuals and identified 185 proteins differentially expressed in these two groups. African American and Hispanic populations have a greater risk of developing AD and non-AD dementia than non-Hispanic white adults; however, these groups are commonly underrepresented in proteomic studies of AD. Even though GluA4 was not differentially expressed between the two ethnicity groups included in this study, these results highlight the need for more research, including ethnically diverse cohorts. This will allow for a better understanding of variabilities in disease-associated phenotypes among different populations so that future therapies can target various ethnic groups.

Overall, the work of Askenazi et al. (2023) [29], compiling studies on AD proteome alterations, postulates that expression of GluA1 and GluA2 is reduced in later stages of AD. Interestingly, this phenotype is not observed in studies of the early stages of the disease, including our study, in which western blot analysis showed no changes in total cell protein expression of GluA1 and GluA2. Since the AD model presented here represents an early stage of the disease, preceding Aβ and tau phenotypes, it can be speculated that abnormalities in AMPAR protein levels have not yet developed at this stage.

In this study, protein expression of GluA3 and GluA4 in iPSC-derived neurons was not analysed, but changes in their expression have been reported in AD *postmortem* brain tissue. GluA3 and GluA4 follow a similar pattern of protein expression as those observed for GluA1 and GluA2. Even though no changes were detected in the early stages of the disease, lower levels of GluA3 were found in the hippocampus, precuneus, frontal, and entorhinal cortex in the late stages of AD pathology [29]. Despite the low number of studies on GluA4, decreased expression of this subunit has been reported in the frontal and entorhinal cortex of advanced stages of AD [86,87]. Thus, future work measuring GluA3 and GluA4 protein levels in day 35 iPSC-derived neurons is required to fully characterise the protein expression of AMPAR subunits in this model. In summary, *postmortem* brain tissue of AD patients shows reduced AMPAR expression, but these changes are only observed in the late/end stages of the disease. Likewise, the results from this study found no differences in total protein levels of GluA1 and GluA2 subunits in FAD iPSC-derived neurons representing early stages of pathology compared to isogenic controls. This suggests the mechanism leading to changes in how FAD neurons respond to AMPA, compared to controls, is not explained by alterations in the total cellular protein expression of these two subunits. Nonetheless, analysis of total cellular protein expression may mask changes in protein expression in specific cellular compartments. The expression of AMPARs in the plasma membrane, specifically, is crucial for receptor function and can be regulated through post-translational modifications. Reversible post-translational modifications, such as phosphorylation, palmitoylation, and ubiquitination, affect receptor subunit exocytosis, endocytosis, degradation, and gating [88]. Phosphorylation of GluA1 regulates synaptic plasticity by potentiating AMPAR responses to glutamate [89] and increasing channel conductance [90] and open probability [91]. While phosphorylation of GluA1 at epitopes S818, S831, and S845 promotes synaptic insertion of AMPAR and dephosphorylation causes endocytosis, dephosphorylation of GluA1 at S567 increases receptor expression at the synapse [88]. GluA2 phosphorylation, on the other hand, is required for AMPAR internalisation in the CA1 region of the hippocampus both in vitro and in vivo [92,93].

Ubiquitination comprises the attachment of a single ubiquitin or polymeric ubiquitin chains to the lysine residues of a substrate protein [94]. All AMPAR subunits can undergo ubiquitination when treated with AMPA [95]. This post-translational modification is calcium-dependent [95,96] and only occurs in receptors present in the plasma membrane [95]. The ubiquitination of AMPAR signals for lysosomal [96,97] or proteasomal degradation [98] of this receptor, and most studies report it also regulates endocytosis [96,98,99]. Importantly, ubiquitination of AMPAR also modulates synaptic transmission [96,98,99,100]. In rat neuronal cultures, AMPA treatment increases the number of internalised GluA1. This phenotype is abolished in GluA1 mutants lacking ubiquitination sites. Instead, mutant GluA1 shows reduced GluA1 degradation and internalisation and increased GluA1 expression at the cell surface [96,98]. Finally, human *postmortem* brain tissue of AD patients showed increased expression of ubiquitinated GluA1 protein, suggesting ubiquitination of AMPAR subunits may play an important role in modulating plasma membrane expression and function. Proteins can also be modified by being covalently bound to lipids, such as fatty acids. This process is termed fatty acylation and includes the post-translational modification palmitoylation, which is the addition of the 16-carbon saturated fatty acid palmitate to one or more intracellular cysteine residues of target proteins. All four AMPAR subunits can be palmitoylated, and they are differentially regulated depending on the site of palmitoylation [101]. In HEK293T cells and primary cortical neurons, palmitoylation of cysteines in the transmembrane domain 2 of GluA1 or GluA2 accumulates these subunits in the Golgi and reduces their surface expression. C-terminal palmitoylation of AMPAR, on the other hand, does not influence steady-state surface expression of this receptor but increases its internalisation after NMDA or AMPA stimulation [101,102]. A knock-in GluA1 C811 palmitoylation-deficient mouse model (GluA1C811S) showed elevated expression of GluA1 in the cortex [103]. Altogether, these studies demonstrate that various post-translational changes to AMPARs influence their trafficking and consequent membrane expression and receptor function. Hence, future work needs to be conducted to measure the expression of all AMPAR subunits intracellularly and in the plasma membrane and to measure post-translational changes that may regulate their trafficking to and from the membrane.

Apart from AMPAR regulation via post-translational modifications, the composition of the lipid membrane, where AMPARs are embedded, provides another layer of ion channel modulation. Presynaptic and postsynaptic membranes are enriched in cholesterol, a sterol lipid that has been linked to AD. Although cholesterol does not affect AMPA binding to its receptor [104], it can still modulate AMPAR activity. Analysis of intracellular calcium levels in cultures of rat hippocampal neurons demonstrated that cholesterol depletion decreased AMPAR-mediated calcium influx [105]. In rat hippocampal slices, cholesterol depletion reduced both the amplitude of AMPAR-mediated excitatory postsynaptic currents (EPSCs) [106] and basal synaptic transmission [105]. Using the same model, it was shown that AMPA treatment potentiated basal synaptic transmission in both normal and cholesterol-depleted conditions. However, in the absence of cholesterol, this potentiation lasted for a shorter period, and the responses to AMPA were not fully abolished after washing out the drug, as was the case for control slices [105]. Nevertheless, contrasting results were found in rat cerebellar granule cells and mouse cortical neurons, where cholesterol depletion reduced NMDA-evoked currents but had no effect on AMPAR-mediated currents [107,108]. This divergence could possibly be explained by the different cell types analysed, the concentration and duration of the stimulus, or the AMPAR agonist utilised by each study. Hence, further investigation is required to understand the effect of neuronal membrane cholesterol content on AMPAR function.

The poly-unsaturated fatty acid arachidonic acid (AA) has also been implicated in neuronal excitability. Mouse brain slices expressing human APP (hAPP) showed higher surface expression of GluA1 and GluA2 subunits of AMPARs when treated with AA, which resulted in increased neuronal activity. AA is generated from the hydrolysis of phospholipids by phospholipase-A2 (PLA_2_), an enzyme that was hyperphosphorylated in hAPP animals, suggesting elevated activation [109]. To confirm this hypothesis, the authors blocked PLA_2_ activity, which prevented the increase in AMPAR protein expression as well as neuronal hyperactivity. PLA_2_ further acts in synaptic transmission by increasing AMPA affinity, binding to its receptor [110], and modulating AMPAR phosphorylation [111]. In rat brain slices, inhibition of calcium-independent PLA_2_ increased GluA1 phosphorylation at residue S831, while inhibition of calcium-dependent PLA_2_ enhanced phosphorylation of GluA2/3 at S880/891 [111]. Although the authors did not investigate the impact of AMPAR phosphorylation on neuronal activity, it is possible that it could affect AMPAR trafficking and expression.

## 5. Future Directions

In this study, iPSC-derived neurons from FAD patients bearing *PSEN1* mutations showed greater calcium responses to glutamate and AMPA than isogenic control neurons. This occurred in the absence of overt Aβ42 and tau S404 phosphorylation phenotypes or changes in total cellular protein expression of GluA1 and GluA2 subunits of AMPAR. Future work should measure total cellular protein expression of GluA3 and GluA4 subunits, as well as expression of all AMPAR subunits in the neuronal membrane and intracellular compartments separately. Trafficking of AMPARs to the membrane can be regulated by post-translational modifications such as phosphorylation, ubiquitylation, and palmitoylation; thus, investigating levels of these post-translational modifications to AMPAR subunits and the enzymes involved in these pathways could demonstrate the mechanisms promoting early calcium signalling dysfunction in FAD neurons. 

## 6. Conclusions

The iPSC-derived cortical excitatory neurons from FAD patients display elevated calcium responses to glutamate and AMPA compared to their isogenic controls. This early calcium phenotype was observed in the absence of an Aβ or tau phenotype. Although mRNA levels of the AMPAR subunits *GRIA1, GRIA2,* and *GRIA4* were increased in FAD1 neurons compared to IC1 neurons, this difference was not significant between FAD2 and IC2 neurons. Furthermore, protein expression of the AMPAR subunits GluA1 and GluA2 was not significantly different between FAD and isogenic control neurons. Hence, the difference in how FAD neurons respond to AMPA cannot be explained by changes in total cellular protein expression of AMPAR subunits GluA1 or GluA2. 

## Figures and Tables

**Table 1:** Details of induced pluripotent cell lines.

iPSC Name	Disease Status	WT/Mutation	APOE Genotype	Age at Collection	Sex	iPSC Line Characterisation
FAD1	Familial AD	*PSEN1* ^S290C^	ε3/3	47	M	[39]
IC1	Isogenic control	*PSEN1* ^WT^	ε3/3	47	M	[39]
HC1	Healthy control	*PSEN1* ^WT^	ε2/4	57	F	[40]
FAD2	Familial AD	*PSEN1* ^A246E^	ε3/4	56	F	[41]
IC2	Isogenic control	*PSEN1* ^WT^	ε3/4	56	F	Anastacio et al., 2024 in revision
HC2	Healthy control	*PSEN1* ^WT^	ε2/3	75	F	[41]

Abbreviations: FAD—Familial Alzheimer’s disease; HC—Healthy control; IC—Isogenic control; iPSC—induced pluripotent stem cell.

**Table 2:** List of primers used for reverse transcription-quantitative polymerase chain reaction (RT-qPCR).

Target	Sequence	Tm
B2M	F: AAGGACTGGTCTTTCTATCTC R: GATCCCACTTAACTATCTTGG	55 °C
GAPDH	F: GAGCACAAGAGGAAGAGAGAGACCC R: GTTGAGCACAGGGTACTTTATTGATGGTACATG	58 °C
HPRT1	F: TGACACTGGCAAAACAATGCA R: GGTCCTTTTCACCAGCAAGCT	58 °C
GRIA1	F: CTAGAAGATCCTTATGTGATGC R: CTCCGTATTTTCCATCACTG	58 °C
GRIA2	F: GGAATCTCCGTATGTTATGATG R: TTGTACTTGAACCCACAATG	55 °C
GRIA3	F: TATTGTATCTGGGGCGTTAC R: TTGAGAACTCAAGAAGGGAG	55 °C
GRIA4	F: GGTACGATAAAGGTGAATGTG R: AAAAGGTCAGCTTCATTCTC	58 °C

Abbreviations: F—forward; R—reverse; Tm—primer melting temperature.

## Data Availability

Data are available upon request.

## Supplementary Materials

The following supporting information can be downloaded at: https://www.mdpi.com/article/10.3390/life14050625/s1, Figure S1: Representative images and timeline of differentiation, Figure S2: Representative images of no primary antibody control for immunostaining from FAD1, IC1 and HC1, Figure S3: Representative images of no primary antibody control for immunostaining from FAD2, IC2 and HC2, Figure S4: Sample description and images of full membranes for Tau-5 antibody and total protein for FAD1/HC1/IC1, Figure S5: Sample description and images of full membranes for Tau-5 antibody and total protein for FAD2/HC2/IC2, Figure S6: Sample description and images of full membranes for p-Tau-s404 antibody and total protein for FAD1/HC1/IC1, Figure S7: Sample description and images of full membranes for p-Tau-s404 antibody and total protein for FAD2/HC2/IC2, Figure S8: Sample description and images of full membranes for GluA1 antibody and total protein for FAD1/HC1/IC1, Figure S9: Sample description and images of full membranes for GluA1 antibody and total protein for FAD2/HC2/IC2, Figure S10: Sample description and images of full membranes for GluA2 antibody and total protein for FAD1/HC1/IC1, Figure S11: Sample description and images of full membranes for GluA2 antibody and total protein for FAD2/HC2/IC2.
