# Supplementary material for: Familial Alzheimer’s Disease Neurons Bearing Mutations in PSEN1 Display Increased Calcium Responses to AMPA as an Early Calcium Dysregulation Phenotype
Source: Life (Basel). 2024 May 12;14(5):625. doi: 10.3390/life14050625 (PMC11123496; doi:10.3390/life14050625)
Supplement: Supplementary file 1 [file life-14-00625-s001.zip › life-2914967-supplementary.pdf]

Supplementary data

Familial Alzheimer's Disease Neurons Bearing Mutations in PSEN1 Display Increased Calcium Responses to AMPA as an Early Calcium Dysregulation Phenotype

Helena Targa Dias Anastacio, Natalie Matosin and Lezanne Ooi

Supp Figure S1.

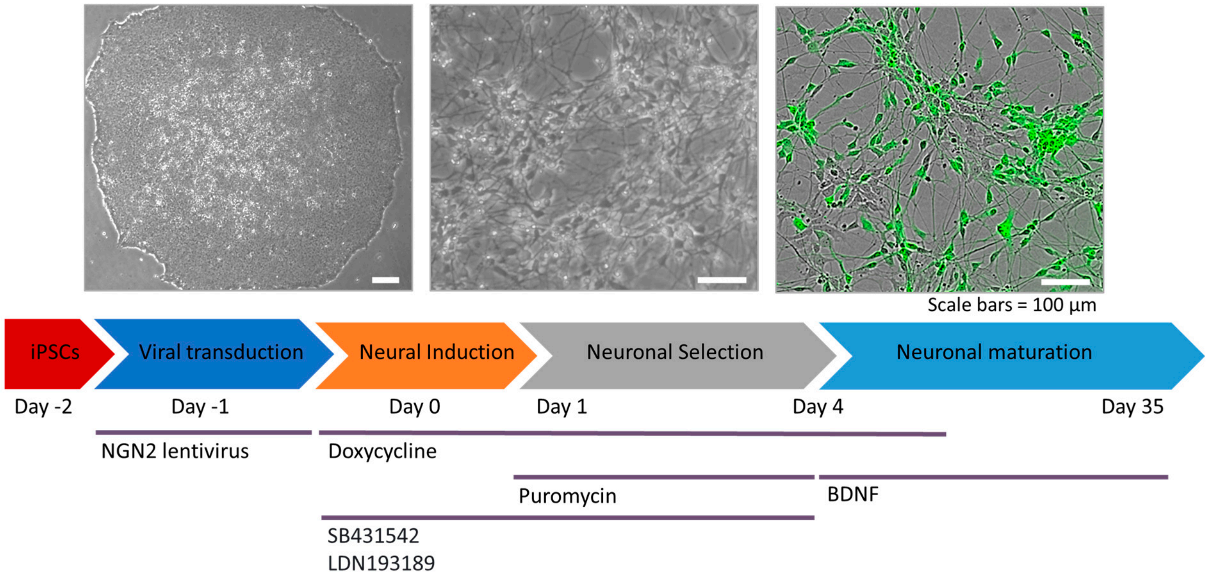

Supp. Figure S1. Representative images and timeline of differentiation. Images show pluripotent stem cell colony and neurons after 35days of differentiation. Expression of GFP confirms expression of the lentiviral Ngn2 construct. Protocol as per Nehme et al., 2018.

Supp. Figure S2.

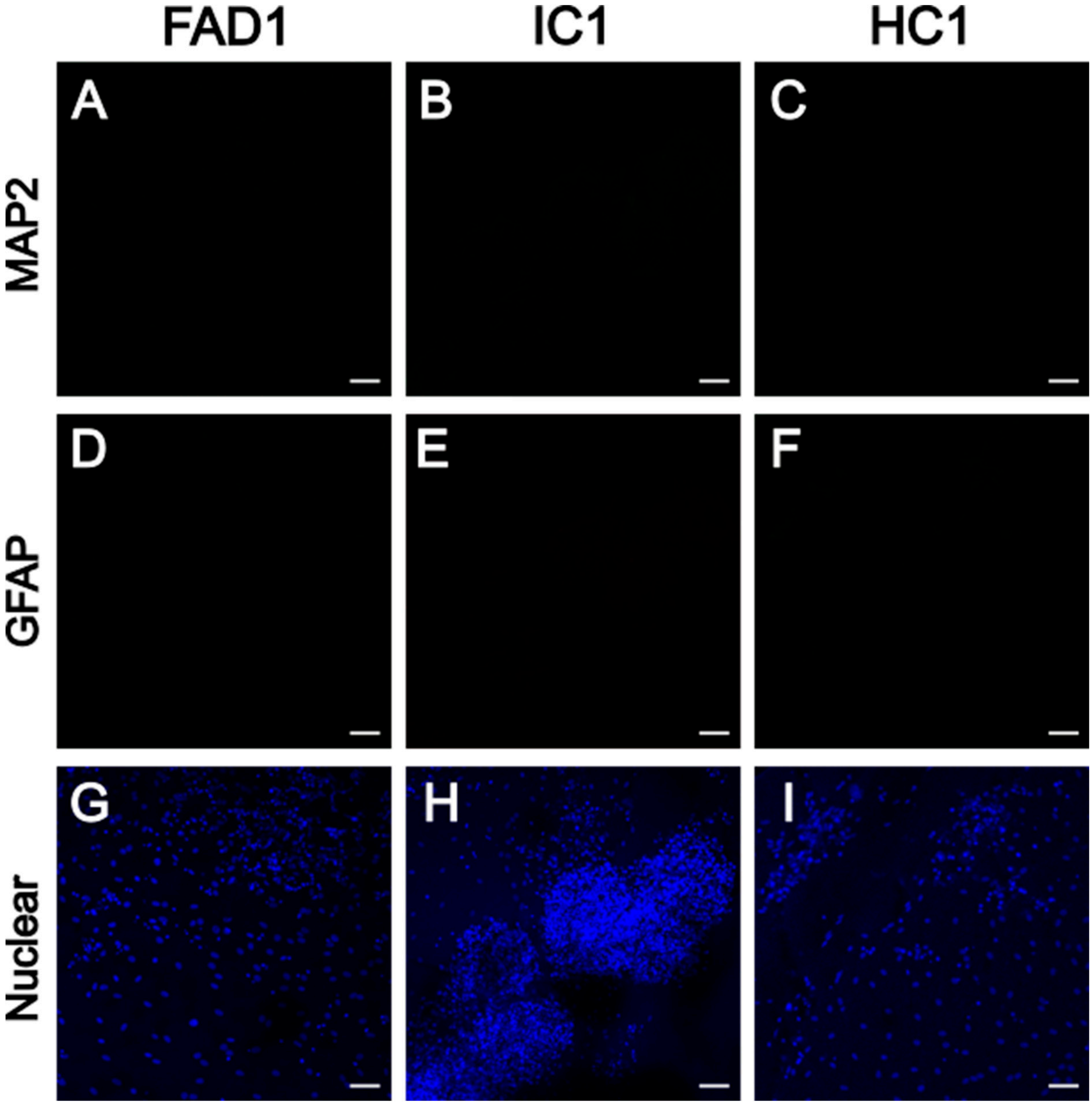

Supp. Figure S2. Representative images of no primary antibody control for immunostaining showing MAP2/green channel (A-C), GFAP/red channel (D-F) and the nuclear marker Hoechst (G-I) from FAD1, IC1 and HC1. Scale bar = 50 µm. FAD: Familial Alzheimer's disease; HC: Healthy control; IC: Isogenic control.

Supp. Figure S3.

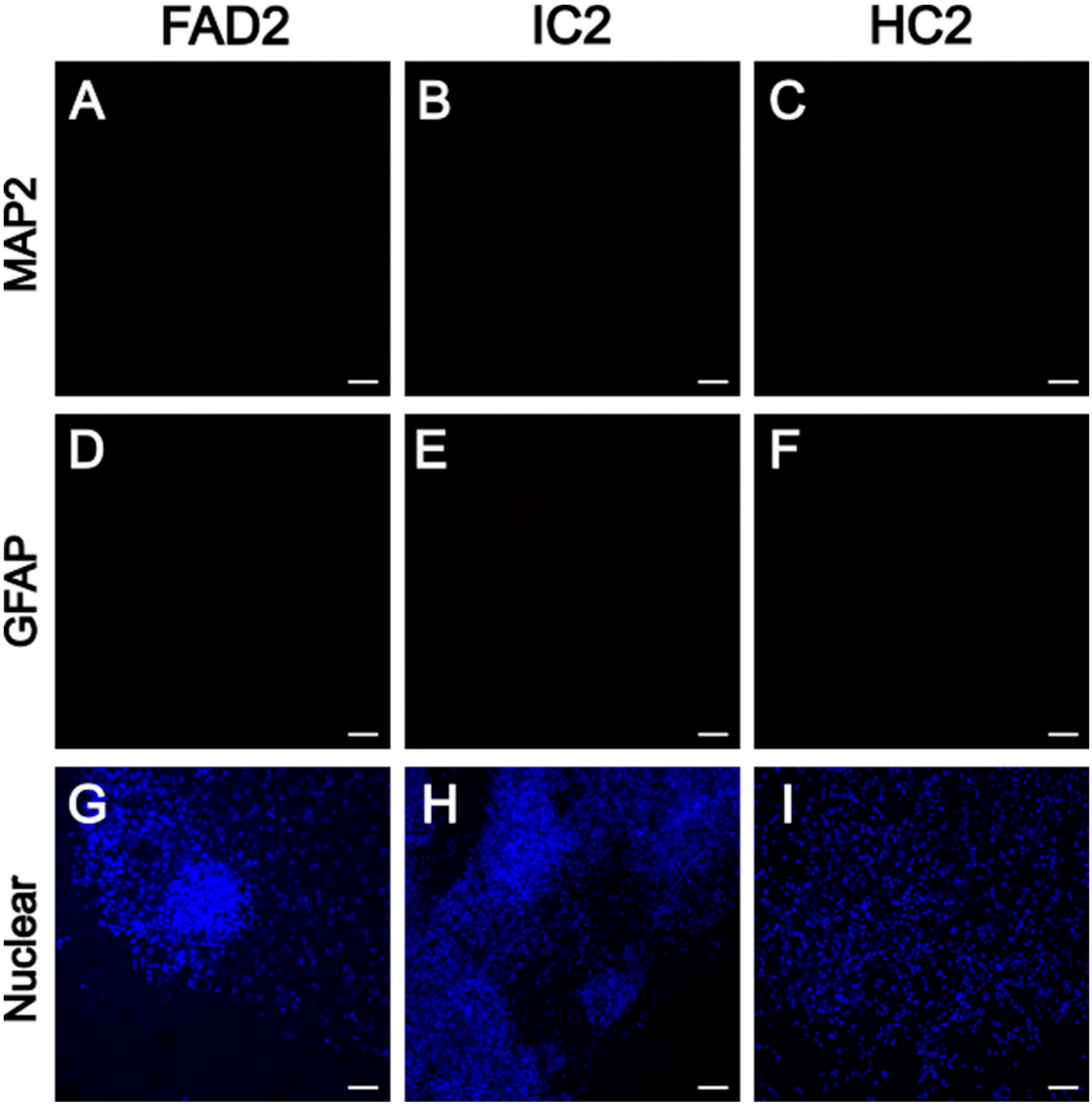

Supp. Figure S3. Representative images of no primary antibody control for immunostaining showing MAP2/green channel (A-C), GFAP/red channel (D-F) and the nuclear marker Hoechst (G-I) from FAD2, IC2 and HC2. Scale bar = 50 µm. FAD: Familial Alzheimer's disease; HC: Healthy control; IC: Isogenic control.

Supp. Figure S4.

Western blot for Tau-5 for FAD1/HC1/IC1

1	Loading buffer
2	mouse brain tissue - NOT INCLUDED
3	Protein marker
4	Loading buffer
5	Loading buffer
6	Pool
7	FAD1.1
8	HC1.1
9	IC1.1
10	FAD1.2
11	HC1.2
12	IC1.2
13	FAD1.3
14	HC1.3
15	IC1.3
16	Pool - NOT INCLUDED
17	FAD1.1
18	HC1.1
19	IC1.1
20	FAD1.2
21	HC1.2
22	IC1.2
23	FAD1.3
24	HC1.3
25	IC1.3
26	Loading buffer

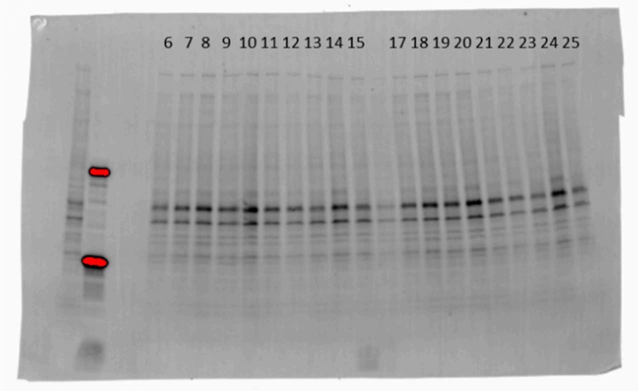

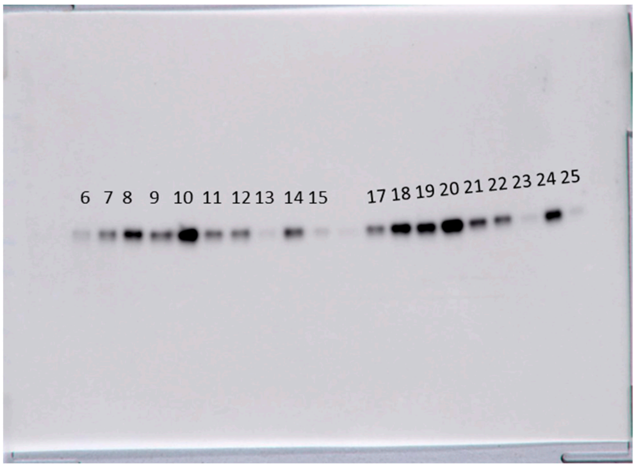

Supp. Figure S4. Sample description and images of full membranes for Tau-5 antibody and total protein for FAD1/HC1/IC1 used for final analysis. All samples were included in the analysis unless specified otherwise. Cell line.1 (e.g. FAD1.1) indicates differentiation replicate 1, 2 or 3. Sometimes blot was imaged without marker (or other lanes) to remove samples that were not quantified, e.g. mouse tissue samples used as positive controls.

Supp. Figure S5.

Western blot for Tau-5 for FAD2/HC2/IC2

extra	mouse brain tissue - NOT INCLUDED
1	Protein marker
2	Loading buffer
3	Pool
4	FAD2.1
5	HC2.1
6	IC2.1
7	FAD2.2
8	HC2.2
9	IC2.2
10	FAD2.3
11	HC2.3
12	IC2.3
extra	Loading buffer

extra	mouse brain tissue - NOT INCLUDED
1	Protein marker
2	Loading buffer
3	Pool
4	FAD2.1
5	HC2.1
6	IC2.1
7	FAD2.2
8	HC2.2
9	IC2.2
10	FAD2.3
11	HC2.3
12	IC2.3
extra	Loading buffer

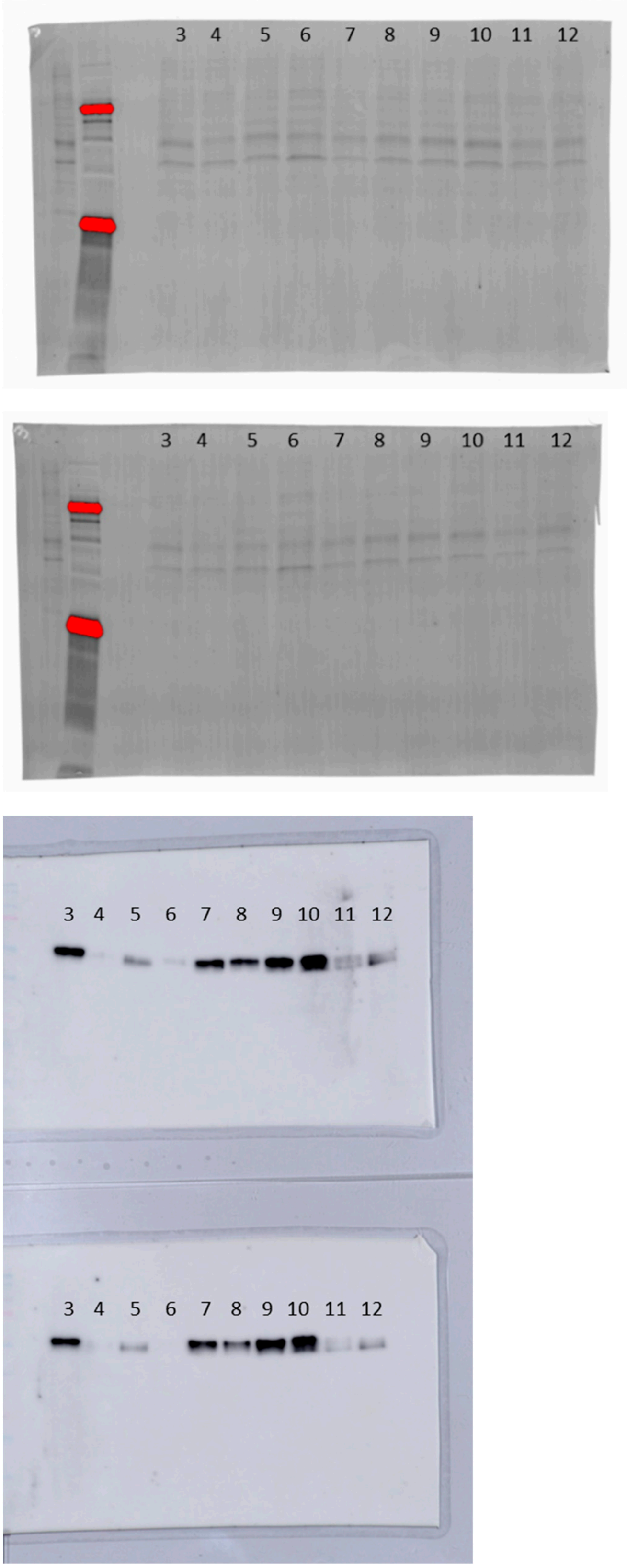

Supp. Figure S5. Sample description and images of full membranes for Tau-5 antibody and total protein for FAD2/HC2/IC2 used for final analysis. All samples were included in the analysis unless specified otherwise. Cell line.1 (e.g. FAD2.1) indicates differentiation replicate 1, 2 or 3. Sometimes blot was imaged without marker (or other lanes) to remove samples that were not quantified, e.g. mouse tissue samples used as positive controls.

Supp. Figure S6.

Western blot for p-Tau-s404 for FAD1/HC1/IC1

1	Loading buffer
2	mouse brain tissue - NOT INCLUDED
3	Protein marker
4	Loading buffer
5	Loading buffer
6	Pool
7	FAD1.1
8	HC1.1
9	IC1.1
10	FAD1.2
11	HC1.2
12	IC1.2
13	FAD1.3
14	HC1.3
15	IC1.3
16	Pool
17	FAD1.1
18	HC1.1
19	IC1.1
20	FAD1.2
21	HC1.2
22	IC1.2
23	FAD1.3
24	HC1.3
25	IC1.3
26	Loading buffer

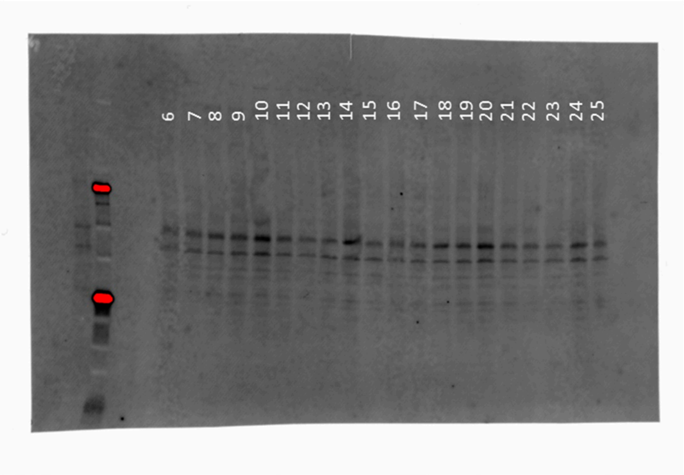

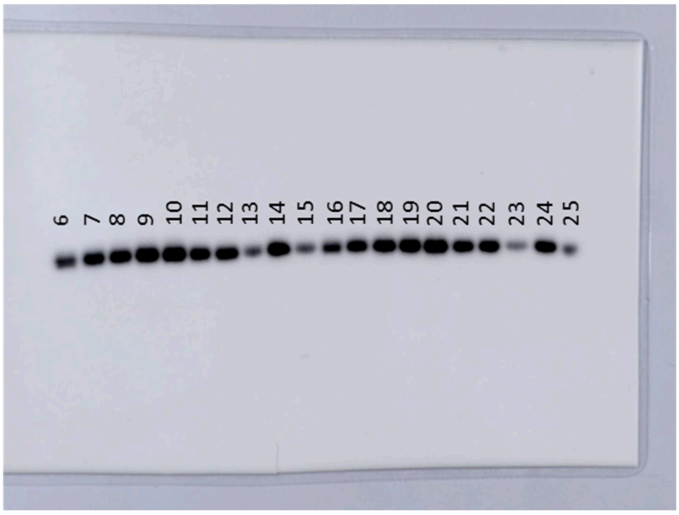

Supp. Figure S6. Sample description and images of full membranes for p-Tau-s404 antibody and total protein for FAD1/HC1/IC1 used for final analysis. All samples were included in the analysis unless specified otherwise. Cell line.1 (e.g. FAD1.1) indicates differentiation replicate 1, 2 or 3. Sometimes blot was imaged without marker (or other lanes) to remove samples that were not quantified, e.g. mouse tissue samples used as positive controls.

Supp. Figure S7.

Western blot for p-Tau-s404 for FAD2/HC2/IC2

extra	mouse brain tissue - NOT INCLUDED
1	Protein marker
2	Loading buffer
3	pool
4	FAD2.1
5	HC2.1
6	IC2.1

7	FAD2.2
8	HC2.2
9	IC2.2
10	FAD2.3
11	HC2.3
12	IC2.3
extra	Loading buffer

extra	mouse brain tissue - NOT INCLUDED
1	Protein marker
2	Loading buffer
3	Pool
4	FAD2.1
5	HC2.1
6	IC2.1
7	FAD2.2
8	HC2.2
9	IC2.2
10	FAD2.3
11	HC2.3
12	IC2.3
extra	Loading buffer

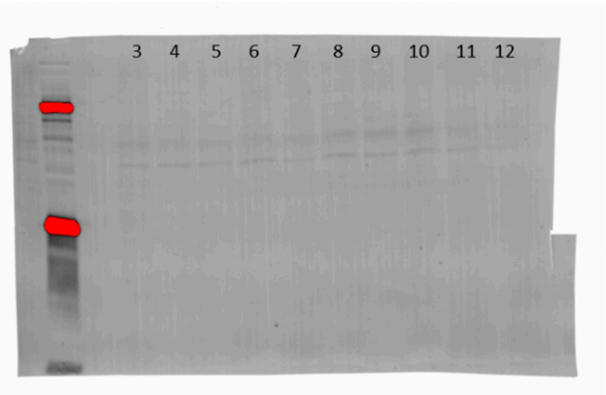

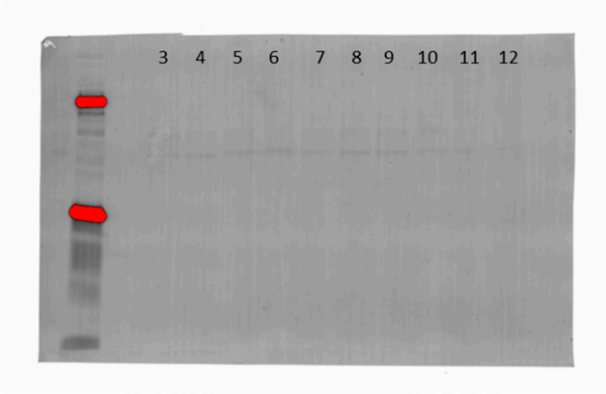

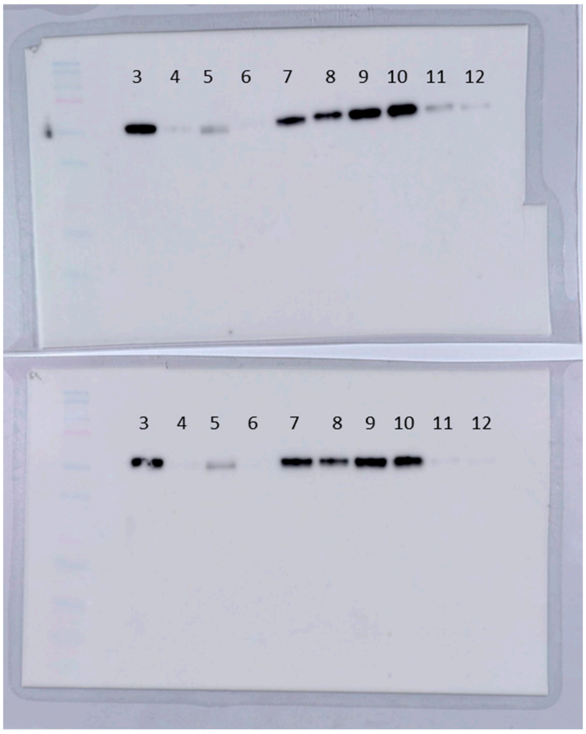

Supp. Figure S7. Sample description and images of full membranes for p-Tau-s404 antibody and total protein for FAD2/HC2/IC2 used for final analysis. All samples were included in the analysis unless specified otherwise. Cell line.1 (e.g. FAD2.1) indicates differentiation replicate 1, 2 or 3. Sometimes blot was imaged without marker (or other lanes) to remove samples that were not quantified, e.g. mouse tissue samples used as positive controls.

Supp. Figure S8.

Western blot for GluA1 for FAD1/HC1/IC1

1	Loading buffer
2	Protein marker
3	mouse brain tissue - NOT INCLUDED
4	Loading buffer
5	Loading buffer
6	Pool
7	FAD1.1
8	HC1.1
9	IC1.1
10	FAD1.2
11	HC1.2
12	IC1.2
13	FAD1.3
14	HC1.3 - NOT INCLUDED
15	IC1.3
16	Pool
17	FAD1.1
18	HC1.1
19	IC1.1
20	FAD1.2
21	HC1.2
22	IC1.2
23	FAD1.3
24	HC1.3 - NOT INCLUDED
25	IC1.3
26	Loading buffer

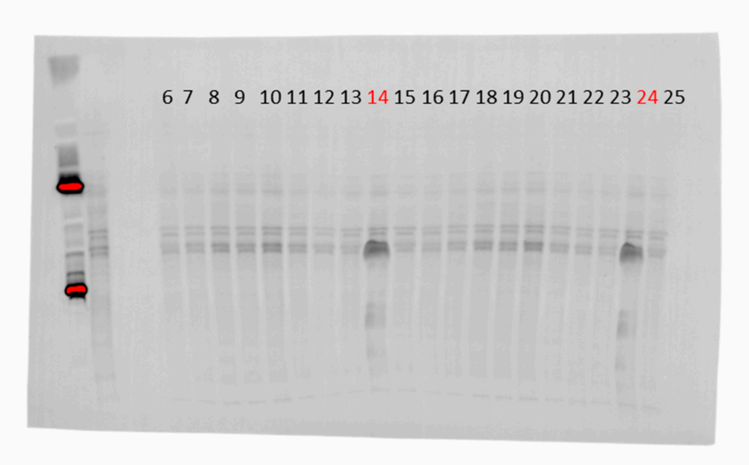

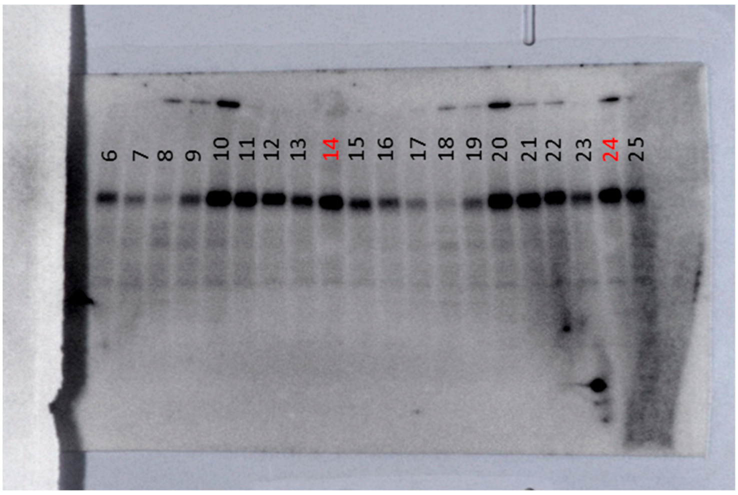

1	mouse brain tissue - NOT INCLUDED
2	Protein marker
3	Loading buffer
4	Pool
5	HC1.3
6	HC1.3
7	Loading buffer

Rest of membrane - NOT INCLUDED

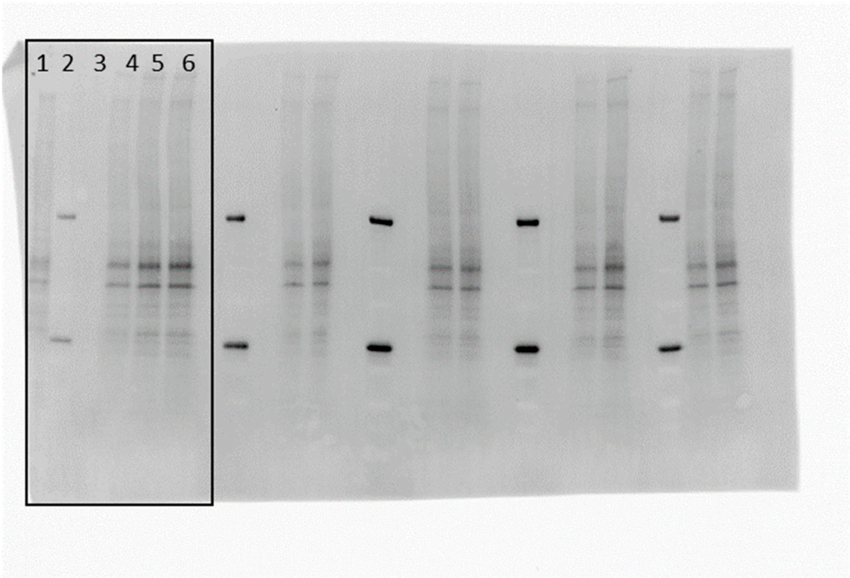

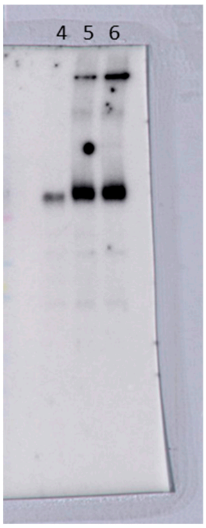

Supp. Figure S8. Sample description and images of full membranes for GluA1 antibody and total protein for FAD1/HC1/IC1 used for final analysis. All samples were included in the analysis unless specified otherwise. Cell line.1 (e.g. FAD1.1) indicates differentiation replicate 1, 2 or 3. Sometimes blot was imaged without marker (or other lanes) to remove samples that were not quantified, e.g. mouse tissue samples used as positive controls.

Supp. Figure S9.

GluA1 for FAD2/HC2/IC2

1	Protein marker
2	Loading buffer
3	Pool
4	FAD2.1
5	HC2.1
6	IC2.1
7	FAD2.2
8	HC2.2
9	IC2.2
10	FAD2.3
11	HC2.3
12	IC2.3
13	Loading buffer
14	mouse brain tissue - NOT INCLUDED

1	Protein marker
2	mouse brain tissue - NOT INCLUDED
3	Loading buffer
4	Pool
5	FAD2.1
6	HC2.1
7	IC2.1
8	FAD2.2
9	HC2.2
10	IC2.2
11	FAD2.3
12	HC2.3
13	IC2.3
14	Protein marker

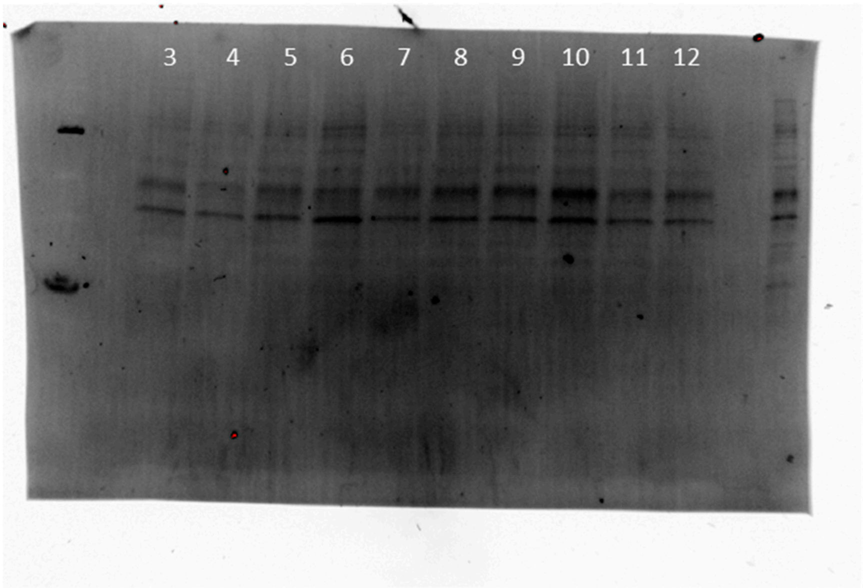
3 4 5 6 7 8 9 10 11 12

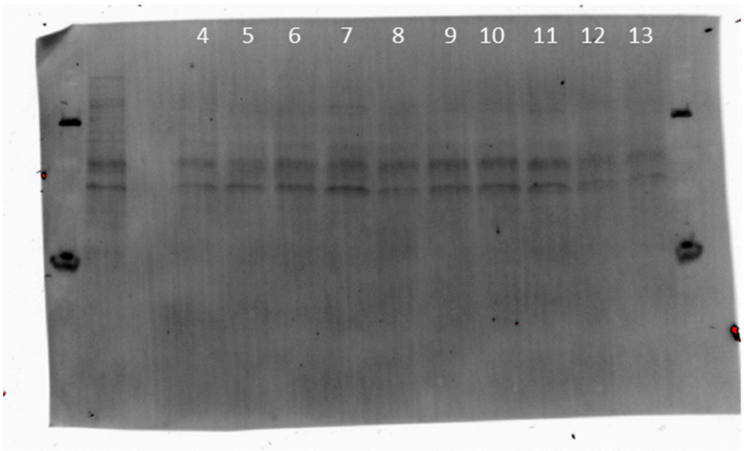
4 5 6 7 8 9 10 11 12 13

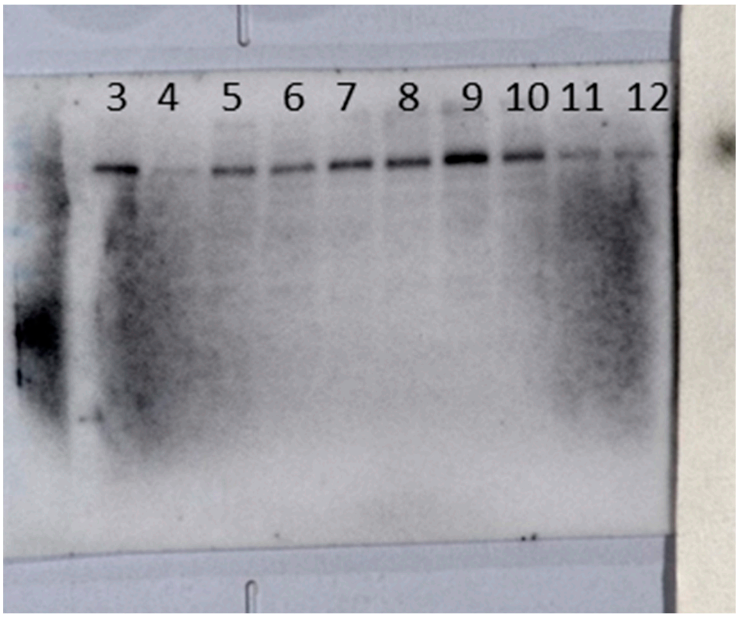
3 4 5 6 7 8 9 10 11 12

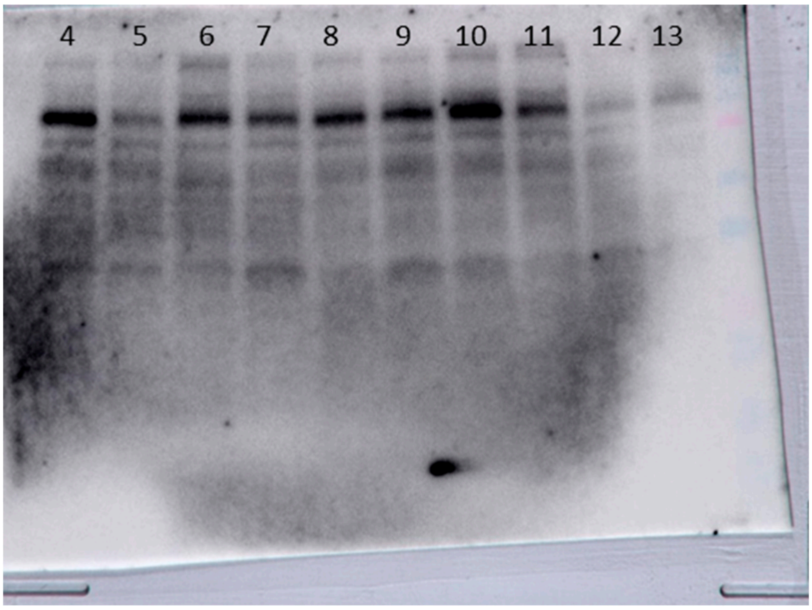

Supp. Figure S9. Sample description and images of full membranes for GluA1 antibody and total protein for FAD2/HC2/IC2 used for final analysis. All samples were included in the analysis unless specified otherwise. Cell line.1 (e.g. FAD2.1) indicates differentiation replicate 1, 2 or 3. Sometimes blot was imaged without marker (or other lanes) to remove samples that were not quantified, e.g. mouse tissue samples used as positive controls.

Supp. Figure S10.

GluA2 for FAD1/HC1/IC1

1	Loading buffer
2	mouse brain tissue - NOT INCLUDED
3	Protein marker
4	Loading buffer
5	Loading buffer
6	Pool
7	FAD1.1
8	HC1.1
9	IC1.1
10	FAD1.2
11	HC1.2
12	IC1.2
13	FAD1.3
14	HC1.3
15	IC1.3
16	Pool - NOT INCLUDED
17	FAD1.1
18	HC1.1
19	IC1.1
20	FAD1.2
21	HC1.2
22	IC1.2
23	FAD1.3
24	HC1.3
25	IC1.3
26	Loading buffer

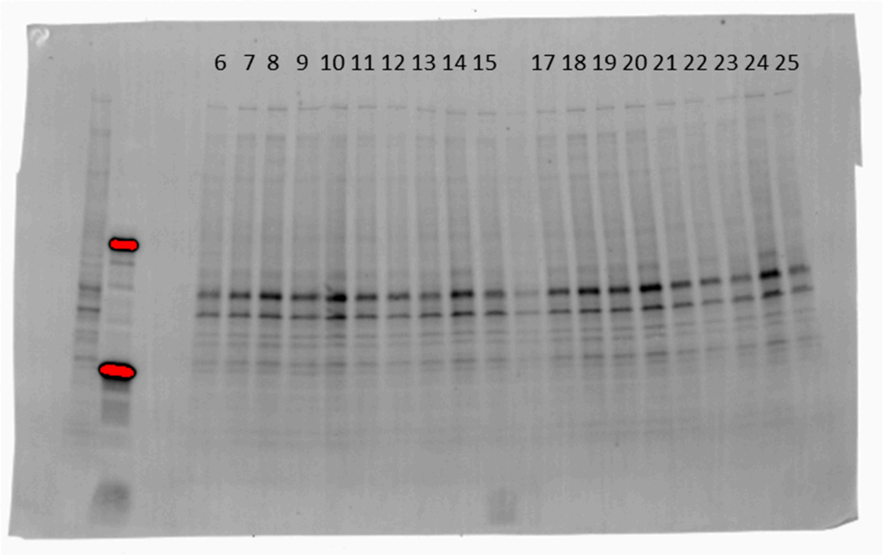

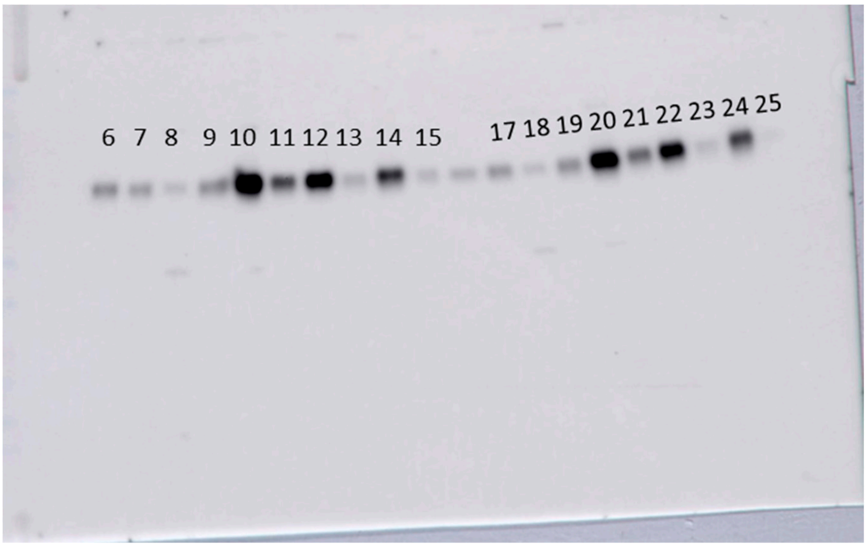

Supp. Figure S10. Sample description and images of full membranes for GluA2 antibody and total protein for FAD1/HC1/IC1 used for final analysis. Same blots as Tau-5. All samples were included in the analysis unless specified otherwise. Cell line.1 (e.g. FAD1.1) indicates differentiation replicate 1, 2 or 3. Sometimes blot was imaged without marker (or other lanes) to remove samples that were not quantified, e.g. mouse tissue samples used as positive controls.

Supp. Figure S11.

GluA2 for FAD2/HC2/IC2

extra	mouse brain tissue - NOT INCLUDED
1	Protein marker
2	Loading buffer
3	Pool
4	FAD2.1
5	HC2.1
6	IC2.1
7	FAD2.2
8	HC2.2
9	IC2.2
10	FAD2.3
11	HC2.3
12	IC2.3
extra	Loading buffer

extra	mouse brain tissue - NOT INCLUDED
1	Protein marker
2	Loading buffer
3	Pool
4	FAD2.1
5	HC2.1
6	IC2.1
7	FAD2.2
8	HC2.2
9	IC2.2
10	FAD2.3
11	HC2.3
12	IC2.3
extra	Loading buffer

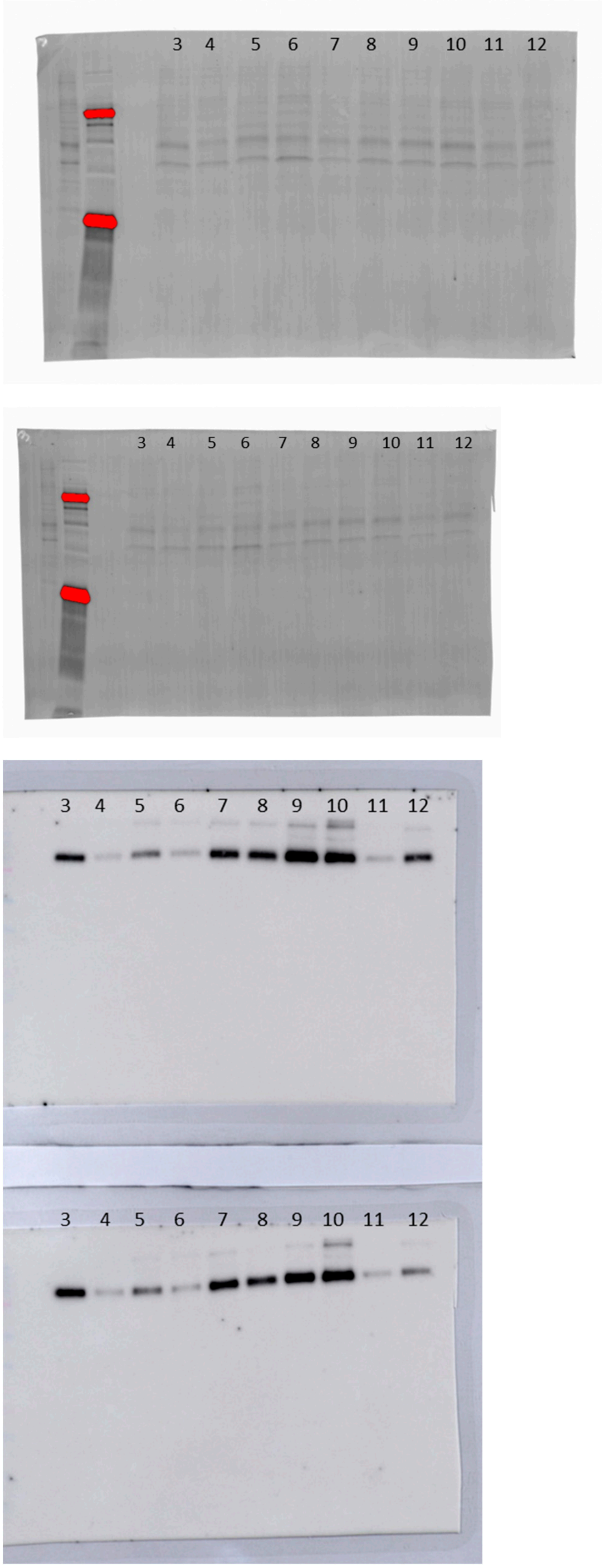

Supp. Figure S11. Sample description and images of full membranes for GluA2 antibody and total protein for FAD2/HC2/IC2 used for final analysis. Same blots as Tau-5. All samples were included in the analysis unless specified otherwise. Cell line.1 (e.g. FAD2.1) indicates differentiation replicate 1, 2 or 3. Sometimes blot was imaged without marker (or other lanes) to remove samples that were not quantified, e.g. mouse tissue samples used as positive controls.
